# Pharmacokinetic Changes According to Single or Multiple Oral Administrations of Socheongryong-Tang to Rats: Presented as a Typical Example of Changes in the Pharmacokinetics Following Multiple Exposures to Herbal Medicines

**DOI:** 10.3390/pharmaceutics13040478

**Published:** 2021-04-01

**Authors:** Seung-Hyun Jeong, Ji-Hun Jang, Da-Hwa Jung, Guk-Yeo Lee, Yong-Bok Lee

**Affiliations:** 1 College of Pharmacy, Chonnam National University, 77 Yongbong-ro, Buk-gu, Gwangju 61186, Korea; rhdqn95@naver.com (S.-H.J.); jangji0121@naver.com (J.-H.J.); 2 Department of Korean Medicinal Resource Development, National Institute for Korean Medicine Development, Udae land gil 288, Jangheung, Jeollanamdo 59338, Korea; jdh7942@nikom.or.kr (D.-H.J.); nara8938@nikom.or.kr (G.-Y.L.)

**Keywords:** pharmacokinetic, Socheongryong-tang, ephedrine, paeoniflorin, cinnamic acid

## Abstract

The purpose of this study was to investigate the pharmacokinetic properties of ephedrine, paeoniflorin, and cinnamic acid after single or multiple doses of Socheongryong-tang (SCRT) were administered to rats, and to present an example of the pharmacokinetic changes following multiple doses of an herbal medicine. SCRT is a traditional herbal medicine that has been used clinically for a long time, and its main ingredients include ephedrine, paeoniflorin, and cinnamic acid. However, studies on the pharmacokinetic properties of SCRT are insufficient, and particularly, no pharmacokinetic information has been reported for multiple doses. In this study, SCRT was administered orally to rats once or multiple times, and plasma sampled at different times was quantitatively analyzed for ephedrine, paeoniflorin, and cinnamic acid using ultra-high-performance liquid chromatography-tandem mass spectrometry. There was a difference between the pharmacokinetic parameter values of each component (especially in paeoniflorin and cinnamic acid) obtained after single or multiple doses of SCRT. The actual observed values of each component obtained after multiple doses of SCRT were clearly different from the predicted results of multiple-dose simulations based on the pharmacokinetic profiles obtained after a single dose. The results confirmed that the plasma concentrations and, thus, exposures to paeoniflorin and cinnamic acid were significantly increased when SCRT was administered multiple times, whereas that of ephedrine was not. The results of this study are expected to provide useful pharmacokinetic data for the safety and efficacy evaluation of SCRT in the future and demonstrate the necessity of pharmacokinetic comparison studies according to single or multiple oral administrations of herbal medicines.

## 1. Introduction

Socheongryong-tang (SCRT), a remedy for respiratory diseases well-known in Korea, Japan, and China, consists of eight herbal medicines: *Ephedrae Herba*, *Paeoniae Radix*, *Asari Herba*, *Zingiberis Rhizoma*, *Glycyrrhizae Radix*, *Cinnamoni Ramulus*, *Pinelliae Rhizoma*, and *Schisandrae Fructus* [1]. SCRT, also known as Sho-Seiryu-to or Xiao-Qing-Long-tang, has been used to treat respiratory diseases such as colds, allergic rhinitis, and bronchial asthma for several centuries in Asian countries [2,3,4]. SCRT has mainly been used for treating allergic rhinitis, allergic asthma, and colds, and is also used for conjunctivitis, eczema, blisters, and nephritis [5]. It was found that SCRT exhibits antihistamine activity, and in addition, it has an extension of bronchial smooth muscle [6]. The clinical use of SCRT is on the rise, and in Korea, SCRT tablets were registered as a health insurance drug in 2016 [7,8].

The main pharmacologically active ingredients of SCRT are ephedrine, paeoniflorin, and cinnamic acid [7,9]. Although, as described above, SCRT is composed of 8 kinds of herbal medicines, so there may be various components in SCRT as follows: Ephedrine (for *Ephedrae Herba*), cinnamic acid (for *Cinnamoni Ramulus*), gingerol (for *Zingiberis Rhizoma*), methyl eugenol (for *Asari Herba*), schizandrin (for *Schisandrae Fructus*), paeoniflorin (for *Paeoniae Radix*), sitosterol (for *Pinelliae Rhizoma*), and glycyrrhizin (for *Glycyrrhizae Radix*) [1]. Nevertheless, the reasons why ephedrine, paeoniflorin, and cinnamic acid were selected as target substances for the pharmacokinetic study of SCRT in this study were as follows: In the content analysis results of the SCRT tablets used in this study, the major contents were ephedrine, paeoniflorin, and cinnamic acid; in addition, access to information on ephedrine, paeoniflorin, and cinnamic acid was most realistically possible in accessing the dose information for each component, which is one of the most important information in pharmacokinetic analysis. In the content analysis results of the provided SCRT tablets, the content was highest in the order of paeoniflorin > ephedrine > cinnamic acid > schizandrin. According to the pharmacokinetic profile obtained after a single oral administration of SCRT 9 tablets to humans in a previous clinical study [7], the maximum plasma concentration of schizandrin was very low, 2.14 ± 1.34 ng/mL, which was lower than the values of paeoniflorin, ephedrine, and cinnamic acid. Furthermore, paeoniflorin, ephedrine, and cinnamic acid were continuously quantified in plasma up to 36 h after a single oral administration of SCRT, but schizandrin was only detected up to 24 h. As a result, as the content of schizandrin in SCRT tablets was relatively low, the degree of quantification in vivo was also limited. Even in a pilot study (data not shown) conducted in small rats prior to this study, the quantification of schizandrin in plasma after the oral administration of SCRT suspension (120 mg/kg) was not confirmed. Therefore, in this pharmacokinetic study, according to single or multiple doses of SCRT, unlike in the past clinical study [7], only the three main components of paeoniflorin, ephedrine, and cinnamic acid, except schizandrin, were selected as the target substances.

Ephedrine is the major active ingredient of *Ephedrae Herba*, with efficacy in vasoconstriction and elevating blood pressure, sweating, diuresis, and sputum removal [10]. Paeoniflorin is the major active ingredient of *Paeoniae Radix* and exerts vasodilation, anti-oxidant, anti-cancer, and anti-inflammatory effects [11]. Cinnamic acid is the main active ingredient of *Cinnamoni Ramulus* and has anti-cancer, anti-bacterial, and anti-oxidant properties similar to those of paeoniflorin [12]. As mentioned, the efficacies of SCRT and its individual components are well known, but pharmacokinetic studies on SCRT are still very scarce. Although SCRT has been used for a long time and is listed in the herbal medicine standard book [1,7], it is an herbal medicine that has been recognized empirically. However, its safety and effectiveness have not been adequately verified scientifically, like other herbal medicines. Thus, pharmacokinetic studies are essential to more efficiently evaluate the safety and efficacy of SCRT. In addition, based on the pharmacokinetic results, the dosage of the drug required to reach blood concentrations within the therapeutic range can be determined [13,14,15]. 

Pharmacokinetic results in humans following a single oral administration of SCRT have been reported [7]. However, there have been no reports on the pharmacokinetic results of multiple doses of SCRT. Traditional herbal medicines such as SCRT tend to be used more than once because of the relatively lower side effects and the chronic use for disease treatment compared to chemical or biological drugs, and medication compliance is relatively high [14,16]. Moreover, for SCRT tablets, the approved clinical regimen is oral administration three times a day [7]. Therefore, studies on the pharmacokinetics of not only a single administration but also multiple administrations of SCRT are essential and required, as for chemical and biological drugs. According to the necessity of such research, a pharmacokinetic study of SCRT administered in single or multiple doses was conducted, and it also intended to show a typical example of pharmacokinetic changes following multiple doses of an herbal medicine. The presentation and comparison of the pharmacokinetic results of single or multiple doses of SCRT as an example of herbal medicine are expected to be useful reference data for clinical applications and safety and efficacy evaluation studies of SCRT in the future.

The novelties of this study were as follows: This study was first to simultaneously evaluate the pharmacokinetics of ephedrine, paeoniflorin, and cinnamic acid after single or multiple oral doses of SCRT to rats. This was a new study focusing on the changes in pharmacokinetics according to single or multiple doses of SCRT (selected as an example of herbal medicines). This study presented valuable and important pharmacokinetic changes (including a comparison with pharmacokinetic results after a single dose) according to multiple exposures (of SCRT) that should be considered very important in other herbal medicines in a situation where the use of herbal medicinal materials is gradually increasing worldwide.

## 2. Materials and Methods

### 2.1. Chemicals and Reagents

**Figure 1:**
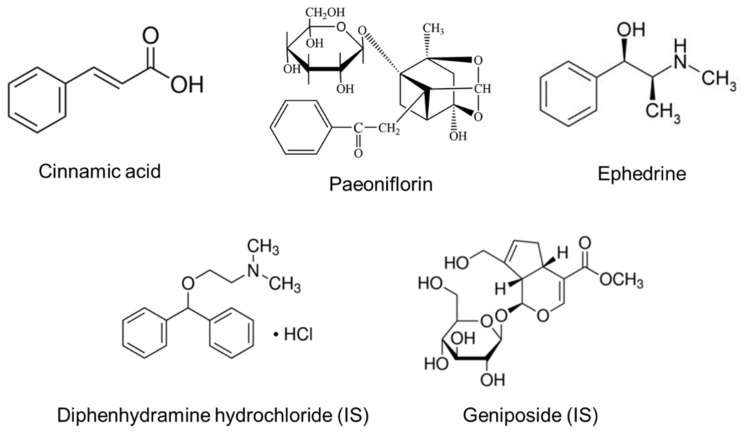
Chemical structures of cinnamic acid, paeoniflorin, and ephedrine as analytes and diphenhydramine hydrochloride and geniposide as internal standards (ISs).

The reference standard for ephedrine (purity ≥ 99%; CAS Number: 299-42-3) was purchased from Sigma-Aldrich (St. Louis, MO, USA). The reference standards for cinnamic acid (purity ≥ 98%; CAS Number: 140-10-3) and paeoniflorin (purity ≥ 98%; CAS Number: 23180-57-6) were obtained from the National Development Institute of Korean Medicine (Jangheung-gun, Jeollanam-do, Republic of Korea). Diphenhydramine hydrochloride (purity ≥ 99%; CAS Number: 147-24-0) and geniposide (purity ≥ 98%; CAS Number: 24512-63-8) as an internal standard (IS) were purchased from Sigma-Aldrich. Figure 1 shows the structure of cinnamic acid, paeoniflorin, ephedrine, and the IS. Liquid chromatography–mass spectroscopy (LC-MS)-grade acetonitrile, methanol, water (18.2 mΩ), and high-performance liquid chromatography (HPLC)-grade ethyl acetate were purchased from Fisher Scientific (Hampton, NH, USA). LC-MS-grade formic acid and acetic acid were supplied from Sigma-Aldrich. All other reagents were in the highest HPLC grades or quality available. Heparin sodium (25,000 IU/5 mL) was purchased from JW Pharmaceutical Corporation (Seoul, Republic of Korea). The SCRT tablets (manufacturing date: 10.01.2017; manufacturing number: H71501502) used in this study were supplied from Kyung-bang Pharmaceutical Company (Incheon, Republic of Korea).

### 2.2. Instrumentation and Chromatographic Conditions

The analysis of ephedrine, paeoniflorin, and cinnamic acid in rat plasma was performed using a Shimadzu Nexera X2 Series ultra-high-performance liquid chromatography (UHPLC) system (Shimadzu Corp., Kyoto, Japan) coupled to an 8040 mass spectrometer (Shimadzu Corp.). The optimized chromatographic separation of ephedrine and cinnamic acid was conducted with a HALO-C_18_ column (100 mm × 2.1 mm, 2.7 μm particle size; Advanced Materials Technology Inc., Wilmington, DE, USA) at a temperature of 40 °C. The optimized chromatographic separation of paeoniflorin was performed on a Kinetex core–shell biphenyl column (50 mm × 4.6 mm, 1.7 μm particle size; Phenomenex Inc., Torrance, CA, USA) at a temperature of 40 °C. In the analysis of ephedrine, the mobile phase was 0.1% (*v*/*v*) of formic acid in water (mobile phase A) and methanol (mobile phase B) with gradient elution, and a flow rate of 0.2 mL/min. The elution program of the mobile phase was 0–0.25 min (5% B), 0.25–1.25 min (5–90% B), 1.25–3.0 min (90% B), 3.0–3.01 min (90–5% B), and 3.01–4.50 min (5% B). Thus, the total run time was 4.5 min, and the injection volume was 5 μL. All analytical procedures were evaluated by positive electrospray ionization (ESI), and quantification was achieved using multiple reaction monitoring (MRM) modes at *m*/*z* 166.20→148.10 for ephedrine and 256.10→167.10 for IS (diphenhydramine). In the analysis of paeoniflorin, the mobile phase consisted of 0.1% (*v*/*v*) formic acid in water (mobile phase A) and acetonitrile (mobile phase B) with gradient elution at a flow rate of 0.3 mL/min. The elution program was 0–1.0 min (10% B), 1.0–2.5 min (10–25% B), 2.5–3.75 min (25% B), 3.75–3.76 min (25–10% B), and 3.76–6.0 min (10% B). Thus, the total run time was 6.0 min, and the injection volume was 5 μL. All analytical procedures were evaluated by negative ESI and quantification was achieved using MRM modes at *m*/*z* 525.20→449.25 for paeoniflorin and 432.90→225.00 for IS (geniposide). In the analysis of cinnamic acid, the mobile phase consisted of 0.005% (*v*/*v*) of formic acid in water (mobile phase A) and acetonitrile (mobile phase B) with a gradient elution, and a flow rate of 0.2 mL/min. The elution program was 0–0.5 min (5–70% B), 0.5–3.5 min (70% B), 3.5–3.51 min (70–5% B), and 3.51–5.0 min (5% B). Thus, the total run time was 5.0 min and the injection volume was 5 μL. All analytical procedures were evaluated by negative ESI and quantification was achieved using MRM modes at *m*/*z* 146.80→103.00 for cinnamic acid and 432.90→225.00 for IS (geniposide). The acquisition and analysis of data were performed using LabSolutions software (Shimadzu Corp.) with a collision energy of 12, 15, and 13 eV for ephedrine, paeoniflorin, and cinnamic acid, respectively. Table 1 presents a summary of the optimized mass spectrometry conditions. In the analyses of all analytes, the desolvation and ion source temperature were 400 and 250 °C, respectively. The drying nitrogen and nebulizing nitrogen gas flow rate were 15 and 3 L/min, respectively. The capillary voltage was 1.88 kV and the dwell time was 100 ms for all analytes.

### 2.3. Preparation of Calibration Curves and Quality Control Samples

Each standard stock solution of ephedrine, paeoniflorin, cinnamic acid, and the IS was weighed accurately and dissolved in methanol at 1 mg/mL prior to preparing working solutions, and was stored at −20 °C. The standard working solutions of ephedrine (concentrations of 5, 20, 50, 100, 500, 2000, and 10,000 ng/mL), paeoniflorin (concentrations of 2, 5, 10, 50, 100, and 200 ng/mL), and cinnamic acid (concentrations of 1, 10, 50, 100, 250, 500, 1000, and 5000 ng/mL) were prepared by diluting the standard stock solutions for cinnamic acid, ephedrine, and paeoniflorin with 100% of methanol. The IS working solutions (diphenhydramine hydrochloride and geniposide at concentrations of 100 ng/mL) were also prepared by diluting the stock solutions with 100% of methanol. Calibration standards were prepared by adding diluted standard working solutions to blank rat plasma to achieve final concentrations of ephedrine ranging from 0.5 to 1000 ng/mL (with 4 ng/mL of diphenhydramine (as the IS)), paeoniflorin ranging from 0.2 to 20 ng/mL (with 10 ng/mL of geniposide (as the IS)), and cinnamic acid ranging from 0.1 to 500 ng/mL (with 10 ng/mL of geniposide (as the IS)). For accuracy and precision, quality control (QC) samples with four concentrations of 0.5, 1, 500, and 800 ng/mL for ephedrine; 0.2, 0.6, 10, and 16 ng/mL for paeoniflorin; and 0.1, 0.3, 80, and 400 ng/mL for cinnamic acid were similarly prepared. The preparation of the calibration standards and QC samples was conducted on the same day as the analyses.

### 2.4. Extraction Procedure

In the analysis of ephedrine, the rat plasma samples were pretreated using methanol for protein precipitation. A rat plasma sample of 50 μL was added to 10 μL of the IS solution (100 ng/mL of diphenhydramine hydrochloride). The mixed sample was added to 190 μL of methanol, vortex-mixed for 10 min, and centrifuged at 13,000× *g* for 10 min. Then, 150 μL of the supernatant organic layer was separated and filtered through 0.45 μm syringe filters (to remove impurities), and 5 μL aliquots were injected for UHPLC-MS/MS analysis. In the analysis of paeoniflorin, the rat plasma samples were pretreated using methanol for protein precipitation. A rat plasma sample of 90 μL was added to 10 μL of IS solution (100 ng/mL of geniposide). The mixed sample was added to 800 μL of methanol, vortex-mixed for 5 min, and centrifuged at 13,000× *g* for 5 min. Then, 850 μL of the supernatant organic layer was dried gently with a centrifugal evaporator with ultrapure nitrogen gas at 50 °C. The dried matter was reconstituted with 50 μL of 20% (*v*/*v*) of methanol in water containing 0.1% (*v*/*v*) of formic acid and vortex-mixed for 5 min. After centrifugation for 5 min at 13,000× *g*, 5 μL aliquots were injected for UHPLC-MS/MS analysis. In the analysis of cinnamic acid, the rat plasma samples were pretreated using mixed organic solvents of a weak acid, ethyl acetate, and methanol for liquid-liquid extraction and protein precipitation. A rat plasma sample of 90 μL was added to 10 μL of IS solution (100 ng/mL geniposide). The mixed sample was added to 1000 μL of acetic acid-ethyl acetate-methanol (1:4:16, *v/v/v*), vortex-mixed for 5 min, and centrifuged at 13,000× *g* for 5 min. Then, 1000 μL of the supernatant organic layer was dried gently with a centrifugal evaporator with ultrapure nitrogen gas at 25 °C. The dried matter was reconstituted with 50 μL of methanol and vortex-mixed for 5 min. After centrifugation for 5 min at 13,000× *g*, 5 μL of aliquots were injected for UHPLC-MS/MS analysis.

### 2.5. Method Validation

The validation of analytical methods was performed with internationally accepted guidelines: Guidance for Industry: Bioanalytical Method Validation by Food and Drug Administration (FDA) [17]. The details of the method validations performed are presented in Table S1.

### 2.6. Animal Experiments

The approval number of the animal experiment was as follows: CNU IACUC-YB-2017-48. It has been thoroughly reviewed and finally approved by the Chonnam National University Animal Experimental Ethics Committee (Gwangju, Republic of Korea). All animal experiments were conducted by the revised Guidelines for Ethical Conduct in the Care and Use of Animals and the rules of Good Laboratory Practice. Sprague-Dawley male rats (7–8 weeks, 235–260 g) were obtained from Damul Science (Daejeon, Republic of Korea). All rats were housed separately in metabolic cages in a ventilated animal room with a controlled relative humidity (50 ± 5%) and temperature (23 ± 1 °C). They were provided free access to water and food and kept on a 12/12 h light/dark cycle. Here, the food supplied to the rats refers to solid feed for rats and was supplied by Dae-Han Biolink (Eumseong-gun, Chungcheongbuk-do, Republic of Korea). All rats were acclimatized for 7 days prior to participation in the test. The rats were fasted overnight before drug administration (to rule-out food effects) with free access to water. To perform the pharmacokinetic study of SCRT tablets, the rats were randomly divided into six groups (*n* = 5). Three groups received a single dose (at 120 mg/kg) of SCRT orally, and the other three groups received multiple oral doses (at 120 mg/kg; from 0 to 120 h in 8 h intervals) of SCRT. Each of the three groups assigned to single or multiple doses of SCRT was randomly assigned to drug-specific analysis groups.

The SCRT tablets were ground using a tablet mill and only the powders that passed through a 45-mesh (pore size less than 355 μm) were suspended in normal saline to prepare a homogeneous suspension. The SCRT suspensions were orally administered to rats using a Zonde (for the rats, Zonde is an injectable syringe for oral administration), and thus, SCRT was administered directly into the stomach. The dosage volume of the SCRT suspensions was 1 mL/kg for both the single and multiple doses. Blood samples of approximately 0.20–0.25 mL were collected from the jugular vein. The preset sampling times were before administration (0 h) and 0.25, 0.5, 0.75, 1, 2, 4, 6, 8, and 12 h after a single oral administration; and 0, 8, 16, 24, 48, 72, 96, 120, 120.25, 120.5, 120.75, 121, 122, 124, 126, 128, and 132 h after multiple oral administrations. In the multiple oral dose group, blood at 0, 8, 16, 24, 48, 72, 96, and 120 h was sampled just before SCRT was administered. The collected blood samples were placed into microtubes (Axygen, Inc., Union City, CA, USA) containing 5 μL of heparin solution to prevent blood clotting. The plasma samples were immediately separated by centrifuging the blood samples at 10,000× *g* for 10 min and stored at −80 °C until further analysis.

### 2.7. Pharmacokinetic Study

The maximum plasma concentration (C_max_) and the time to reach C_max_ (T_max_) were individually determined by the plasma concentration–time profile. The area under the plasma concentration-time curve (AUC) from 0 to t h after administration (AUC_0–t_) was calculated by the linear trapezoidal rule. The AUC from 0 to infinity (AUC_0–∞_) was calculated as AUC_0–t_ + C_t_/k, where k and C_t_ are the elimination rate constant at the terminal phase and the last measurable concentration, respectively. The clearance (CL/F) and volume of distribution (V_d_/F) were calculated as dose/AUC_0–∞_ and dose/k·AUC_0–∞_, respectively, where F is the bioavailability of oral administration. The half-life (t_1/2_) was calculated as 0.693/k. All analysis results are presented as the mean ± standard deviation (SD). The accumulation ratio (R) was calculated by 1 / (1−e ^−k·τ^), where τ is the dosing interval (as 8 h). All pharmacokinetic parameters were analyzed by WinNonlin^®^ software (version 8.2, Pharsight^®^, a Certara™ Company, Menlo Park, CA, USA) using noncompartmental analysis. Simulations for each component of the multiple doses were performed based on the pharmacokinetic patterns obtained after a single dose of SCRT using the nonparametric superposition option in the same software. This means performing a repeated-dose simulation based on a linear pharmacokinetic system for each component. A schematic diagram summarizing the experimental design is presented as Supplementary data in Figure S1.

### 2.8. Statistical Analysis

The Statistical Package for the Social Sciences (SPSS) software (version 26, IBM, Armonk, NY, USA) was used for the statistical analyses. Statistical significance using the Student’s t-test was applied to all data. Here, *p* < 0.05 indicates a significant difference. The Student’s t-test was mainly used to compare differences in the pharmacokinetic parameters obtained after single or multiple doses of SCRT.

## 3. Results and Discussion

### 3.1. Method Application

In this study, sensitive and accurate UHPLC–MS/MS methods were applied for the quantitative analyses of ephedrine, paeoniflorin, and cinnamic acid in rat plasma. The applied UHPLC-MS/MS methods were optimized for this study by referring to the analysis methods for each component previously reported [18,19,20]. Although there was a simultaneous quantitative analysis of ephedrine, paeoniflorin, and cinnamic acid reported in the past [7], the reason we analyzed each component individually in this study was due to a problem with the sensitivity of cinnamic acid. In the previous simultaneous quantitative analysis report [7], the lower limits of quantitation (LLOQ) of ephedrine, paeoniflorin, and cinnamic acid were 0.5, 0.2, and 1 ng/mL, respectively. In that study [7], the plasma concentrations of cinnamic acid at the times corresponding to the drug elimination phase were very low, approximately 1 ng/mL. Therefore, in this pharmacokinetic study, it was necessary to apply a more sensitive method for cinnamic acid. As a result, the application of a sensitive cinnamic acid analysis method (with a LLOQ of 0.1 ng/mL) reported in the past [18] was inevitable in this study. Therefore, in consideration of sensitivity in this study, an individual analysis for each component was applied instead of the simultaneous quantitative analysis method reported in the past [7]. The reason why the sensitivity of cinnamic acid was improved in the individual analysis compared to the case of simultaneous analysis was due to the optimization of each condition for the analysis of one component (cinnamic acid), as described in detail in a previously published manuscript [18].

The product ion mass spectra of ephedrine, paeoniflorin, and cinnamic acid were obtained using the scan mode of the individual standard solutions in the mass spectrometer. Among the positive and negative ESI modes, paeoniflorin and cinnamic acid generated deprotonated molecular ions such as [M+HCOO−H]^–^ and [M−H]^–^ in the negative-ionization mode. In contrast, ephedrine generated the protonated molecular ion [M+H]^+^ in the positive-ionization mode. The scan mode was performed in the same way for the IS, where diphenhydramine, the IS of ephedrine, produced the protonated molecular ion [M+H]^+^ in the positive-ionization mode. Geniposide, the IS of paeoniflorin and cinnamic acid, produced the deprotonated molecular ion [M+HCOO−H]^–^ in the negative-ionization mode. The most abundant fragment ion for MRM was *m*/*z* 166.2→148.1 for ephedrine, 525.2→449.25 for paeoniflorin, 146.8→103.0 for cinnamic acid, 256.1→167.05 for diphenhydramine, and 432.9→225.0 for geniposide. Figure S2 presents the relevant mass spectra. Various mobile phase compositions, different columns, and organic solvents including extraction and precipitation solvents were tested to determine the optimal separation of each substance. Table S2 summarizes the test results under the above conditions performed for the optimal separation of ephedrine, paeoniflorin, and cinnamic acid. Based on the results of several of these tests shown in Table S2, each substance was isolated and quantified according to the analytical conditions described in Section 2.2 and Section 2.4. In the separation of ephedrine, paeoniflorin, and cinnamic acid, the mobile phases were used in a gradient system. Gradient elution was more satisfactory for the peak shape, retention time, and dissolution than isocratic elution. Figure S3 shows the representative MRM chromatograms with retention times of 2.37 min for ephedrine (with 2.57 min for diphenhydramine as the IS), 3.32 min for paeoniflorin (with 2.88 min for geniposide as the IS), and 2.42 min for cinnamic acid (with 2.17 min for geniposide as the IS) under the optimized conditions.

### 3.2. Method Validation

In this study, the UHPLC-MS/MS methods for ephedrine, paeoniflorin, and cinnamic acid were fully validated in accordance with the FDA Guidance for Industry: Bioanalytical Method Validation (according to the methods presented in Table S1). The representative chromatograms of ephedrine, paeoniflorin, and cinnamic acid are shown in Figures S4–S6, respectively, and there was no significant interference from the endogenous substances around the retention times of the analytes in blank rat plasma. In the rat plasma samples, the intra- and inter-batch precision of the assays was within 9.19% and 8.21%, respectively (Table S3). In addition, the intra- and inter-batch accuracies of the analysis methods were within the ranges of 92.00–110.87% and 90.81–106.69%, respectively (Table S3). The ephedrine, paeoniflorin, and cinnamic acid recovery values from the rat plasma samples were in the ranges of 73.93–76.49%, 82.45–84.81%, and 77.18–81.46%, respectively (Table S4). The matrix effect results of ephedrine, paeoniflorin, and cinnamic acid in the rat plasma samples were within the ranges of 95.96–100.24%, 97.40–100.44%, and 98.77–101.29%, respectively (Table S4). The stability evaluation results of ephedrine, paeoniflorin, and cinnamic acid in rat plasma samples under various conditions such as short-term, long-term, storage, freeze-thaw, and autosampler conditions were within the ranges of 93.33–102.00%, 93.68–102.52%, and 98.53–103.83%, respectively (Table S5). The stability of all standard stock and working solutions for ephedrine, paeoniflorin, and cinnamic acid ranged from 96.41 to 100.28% (Table S6). In the rat plasma sample, the linearities of ephedrine (from 0.5 to 1000 ng/mL), paeoniflorin (from 0.2 to 20 ng/mL), and cinnamic acid (from 0.1 to 500 ng/mL) were confirmed by correlation coefficients of >0.99. The dilution integrity test confirmed that diluting the samples in the same matrix as rat plasma did not significantly affect the precision and accuracy (within ± 15%) of the ephedrine, paeoniflorin, and cinnamic acid analyses. As a result, all of the validation results of the assays were acceptable according to the FDA guidelines [17].

### 3.3. Pharmacokinetic Study

In this study, the reason SCRT was administered orally to rats was that the only route of administration approved in the clinical trial was the oral route. The reason why SCRT is only permitted orally in clinical practice is probably because the development of intravenous formulations has not yet been completed. That is, because the safety of the intravenous administration of SCRT has not been sufficiently secured, the approval for this route of administration has not yet been made. In addition, as far as we know, not only SCRT but also most other herbal medicines are administered orally. This is probably because the efficacy and safety of different routes of administration of herbal medicines have not been sufficiently established. Therefore, it will be necessary to explore additional routes of administration and develop formulations in consideration of the safety and effectiveness of other herbal medicines including SCRT in the future. The rat oral dose was calculated as the ratio of rat-to-human body weight based on the maximum daily dose of 7.2 g in humans. It was established that 120 mg/kg of SCRT could be administered to rats based on the administration of 7.2 g to an approximate average weight of 60 kg for humans. The contents of the three components per three SCRT tablets (test number: QPO-17011001; information provided by Kyung-bang Pharmaceutical Company) were 9.8 mg of ephedrine, 49.5 mg of paeoniflorin, and 0.25 mg of cinnamic acid. As 120 mg/kg of SCRT was administered to the rats, the average doses of ephedrine, paeoniflorin, and cinnamic acid administered to the rats were 0.49, 2.475, and 0.0125 mg/kg, respectively. The dosage information for each of these ingredients was applied to calculate the pharmacokinetic parameters. No visible side effects resulted from administration to the rats. All rats were healthy enough during the test, and the dietary intake did not change significantly from before the test. This implied that the oral administration of 120 mg/kg SCRT was not significantly harmful to the rats and was not a toxic dose.

**Figure 2:**
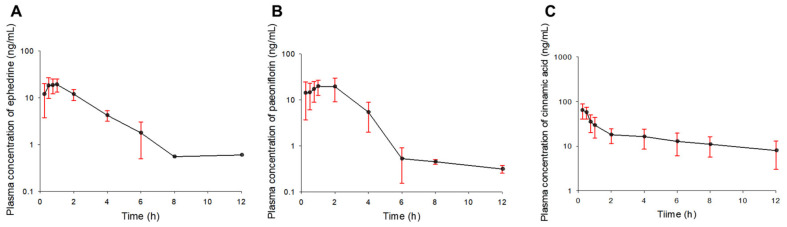
Mean plasma concentration–time profiles of ephedrine (**A**), paeoniflorin (**B**), and cinnamic acid (**C**) obtained after the single oral administration of Socheongryong-tang (SCRT) to rats (*n* = 5). The vertical bars represent standard deviations.

The plasma concentration–time profiles of ephedrine, paeoniflorin, and cinnamic acid according to a single oral administration of SCRT are shown in Figure 2. Ephedrine, paeoniflorin, and cinnamic acid were quantified in plasma up to 12 h after SCRT administration. The average plasma concentrations of ephedrine, paeoniflorin, and cinnamic acid gradually decreased until 8–12 h after the oral absorption process (after SCRT administration). The plasma concentration differences in ephedrine, paeoniflorin, and cinnamic acid were relatively large (with high SD) between rats at the specific times sampled, such as 0.25 and 6 h after the administration for ephedrine; 0.25, 2, 4, and 6 h after the administration for paeoniflorin; and 4, 6, 8, and 12 h after the administration for cinnamic acid. These plasma concentration–time profile patterns of paeoniflorin and cinnamic acid with large differences between individuals may be related to the elution and dissolution of ingredients from SCRT administered to the rats. The SCRT tablets were crushed with a tablet mill, and only powders that passed through a 45-mesh were suspended in normal saline and administered to the rats. Nonetheless, the nonuniform particle size of the powders and the resulting solubility differences may have influenced the pharmacokinetic profiles. In other words, powders with a relatively large particle size have a low solubility, and the components may have been eluted from the gastrointestinal tract later.

**Figure 3:**
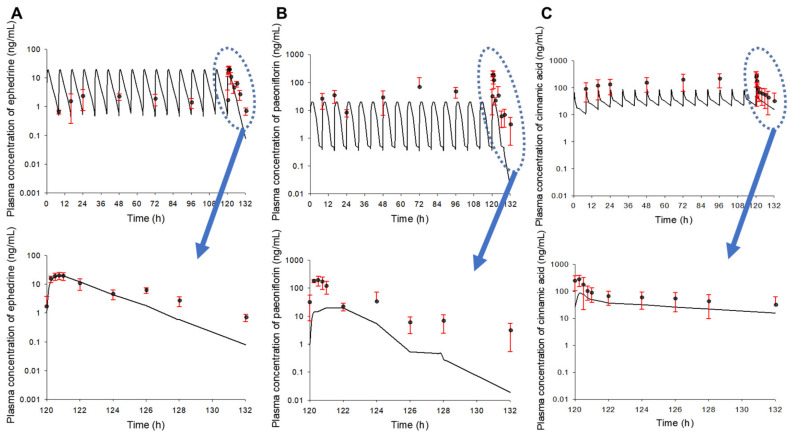
Mean plasma concentration–time profiles of ephedrine (**A**), paeoniflorin (**B**), and cinnamic acid (**C**) obtained after multiple oral administrations (from 0 to 120 h at 8 h intervals) of SCRT to rats (*n* = 5). The solid-line graphs are the simulation (mean value) graphs of multiple doses based on the single-dose (mean) data of SCRT. The black dots represent experimental observation values. The vertical bars represent standard deviations.

The plasma concentration–time profiles of ephedrine, paeoniflorin, and cinnamic acid according to multiple oral administrations of SCRT are shown in Figure 3. In the case of ephedrine, the results of multiple doses simulated from the pharmacokinetic profile obtained after a single dose of SCRT and the visual inspection observations did not differ significantly. Specifically, it showed very good agreement from 120 to 124 h. In contrast, in the case of paeoniflorin and cinnamic acid, there was a large difference between the visual observations and the results of multiple-dose simulations using the pharmacokinetic profile obtained after a single dose of SCRT.

**Figure 4:**
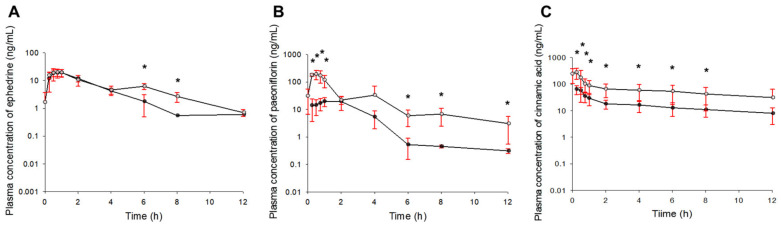
Mean plasma concentration–time profiles of ephedrine (**A**), paeoniflorin (**B**), and cinnamic acid (**C**) obtained after single (black-filled circles; from 0 to 12 h after dosing) or multiple (white-filled circles; from 120 to 132 h after dosing) oral administrations of SCRT to rats (*n* = 5). The vertical bars represent standard deviations. * *p* < 0.05 between the single and multiple administration groups.

The comparison of the plasma concentration–time profiles of ephedrine, paeoniflorin, and cinnamic acid according to single or multiple oral administrations of SCRT is shown in Figure 4. Here, the profiles of multiple oral administrations were observed up to 132 h after the last administration of SCRT at 120 h. Overall, the plasma concentrations of ephedrine, paeoniflorin, and cinnamic acid were higher in multiple oral SCRT administrations than in single oral administrations, and there was no significant difference in the profile patterns between the administration groups. The average plasma concentrations of ephedrine, paeoniflorin, and cinnamic acid gradually decreased until 8–12 h after the oral absorption process (after SCRT administration). In the case of ephedrine, significant differences were shown in the plasma concentration values only 6 and 8 h after SCRT administration, and there was no significant difference in the remaining points between the SCRT single and multiple oral administration groups. In contrast, in the case of paeoniflorin and cinnamic acid, the plasma concentration values were significantly different between the SCRT single and multiple oral administration groups at most time points after SCRT administration.

The pharmacokinetic parameters of ephedrine, paeoniflorin, and cinnamic acid estimated after the single oral administration of SCRT to rats are presented in Table 2. The average T_max_ values of ephedrine, paeoniflorin, and cinnamic acid after the single oral administration of SCRT were less than 1.5 h, suggesting the rapid absorption into the blood. For cinnamic acid, the calculated AUC_0–t_ was lower than the 80% value of the estimated AUC_0–∞_. This may be related to the plasma concentration-time profile of cinnamic acid (Figure 2). According to the plasma concentration–time profile of cinnamic acid obtained after the single administration of SCRT, the concentration of cinnamic acid 12 h after administration was relatively high, at around 10 ng/mL. In addition, the concentrations of cinnamic acid in plasma were gradually eliminated with a low gradient from 2 h after administration to 12 h, which was the last sampling point. Therefore, the t_1/2_ and AUC_t-∞_ (as C_t_/k) in the elimination phase were estimated to be relatively high and the CL/F was estimated to be low. It may also be related to the sensitivity of the cinnamic acid assay applied in this study. The LLOQ for cinnamic acid was very low, at 0.1 ng/mL. Therefore, based on the results of this study, it will be necessary to sample up to 12 h or more, such as 36 or 48 h, after SCRT administration in future pharmacokinetic studies of cinnamic acid following the oral administration of SCRT because the plasma concentration of cinnamic acid was maintained at a relatively high concentration for a considerable period of time. The R values estimated from the elimination rate constants of ephedrine, paeoniflorin, and cinnamic acid calculated after the single oral administration of SCRT were all greater than 1. This indirectly predicted that when SCRT is orally administered multiple times, the exposure concentration of the plasma components would be greater than the simple simulation based on the results of a single administration.

In this study, the pharmacokinetic parameters of ephedrine, paeoniflorin, and cinnamic acid calculated after the single oral administration of SCRT were not significantly different from the previously reported pharmacokinetic results. Table S7 shows the pharmacokinetic parameters obtained after the single oral administration of other herbal medicines or single standard products other than SCRT to rats, and there were differences in the dosage for each ingredient. Therefore, a comparison with previous reports relevant to this study was necessary. The mean T_max_ and t_1/2_ of ephedrine reported in previous studies were 0.29–3.08 and 1.00–9.76 h, respectively [21,22,23,24,25]. The T_max_ and t_1/2_ of ephedrine obtained after the oral administration of SCRT were 0.85 ± 0.22 and 1.62 ± 0.74 h, respectively, within the range of the previously reported values. The mean T_max_ and t_1/2_ of paeoniflorin reported in previous studies were 0.08–2.70 and 0.83–6.94 h, respectively [26,27,28,29,30,31,32,33,34,35,36,37]. The T_max_ and t_1/2_ of paeoniflorin obtained after the oral administration of SCRT were 1.40 ± 0.55 and 1.27 ± 0.55 h, respectively, within the range of the previously reported values. The mean T_max_ and t_1/2_ of cinnamic acid reported in previous studies were 0.08–1.48 and 0.33–4.14 h, respectively [21,38,39,40,41,42,43,44]. The T_max_ of cinnamic acid obtained after the oral administration of SCRT was 0.35 ± 0.14 h, within the range of the previously reported values. However, the t_1/2_ of cinnamic acid obtained after the single oral administration of SCRT was 9.30 ± 8.31 h, which was greater than the range of previously reported values. This was probably due to the use of SCRT suspension, which originated from the tablets, a formulation different from those in previous reports. The t_1/2_ of cinnamic acid after the oral administration of SCRT tablets was estimated to be quite long, at 16.50 ± 6.67 h, in a related previous report [18], although it was a pharmacokinetic study in humans.

According to a report on the pharmacokinetics of SCRT, results have been achieved on healthy humans [7]. According to this report [7], the estimated CL/F and V_d_/F of ephedrine, paeoniflorin, and cinnamic acid in humans (after the oral administration of SCRT tablets) were 19.31–2864.05 L/h and 100.37–16104.13 L, respectively. The estimated CL/F and V_d_/F of ephedrine, paeoniflorin, and cinnamic acid in humans were significantly greater than those in rats (CL/F of 0.05–42.52 L/h/kg and V_d_/F of 0.49–74.05 L/kg). In addition, after the oral administration of SCRT, the T_max_ values of ephedrine, paeoniflorin, and cinnamic acid were shorter in rats (of 0.35–1.40 h) than in humans (of 0.49–3.69 h). For t_1/2_ values, ephedrine and paeoniflorin were shorter in rats (of 1.27–1.62 h) than in humans (of 3.39–5.58 h). The differences in the values of these pharmacokinetic parameters are probably strongly related to differences between species (human versus rat) and the formulations administered in each study. That is, in the past human studies, nine tablets of SCRT were administered, but in this study, a form made of SCRT as a suspension was administered. Therefore, the difference in dissolution and solubility of the ingredients depending on the formulation may cause a major difference in the absorption process in the body.

The pharmacokinetic parameters for ephedrine, paeoniflorin, and cinnamic acid estimated after multiple oral administrations of SCRT to rats are presented in Table 3. There were significant differences in the values of some pharmacokinetic parameters of paeoniflorin and cinnamic acid obtained after single or multiple oral administrations of SCRT. In contrast, in the estimated pharmacokinetic parameter values of ephedrine, there was no significant difference between the SCRT single-oral-dose and the multiple-dose administration groups. The t_1/2_ of paeoniflorin was significantly (*p* < 0.05) increased and the CL/F was decreased from multiple doses compared to a single dose of SCRT. These results suggest that multiple doses of SCRT would increase the level of exposure to paeoniflorin in the blood. The C_max_ values of paeoniflorin and cinnamic acid were significantly (*p* < 0.05) increased and the T_max_ values were decreased from multiple doses compared to a single dose of SCRT. This also implies that the blood exposure of paeoniflorin and cinnamic acid from multiple doses of SCRT would be increased compared to that of a single dose and the significantly (*p* < 0.05) lower CL/F of cinnamic acid calculated after multiple doses of SCRT compared to a single dose would also support this. Compared to other ingredients, the CL/F of paeoniflorin in multiple doses of SCRT was significantly reduced (about 6-fold) compared to that of the single dose. That is, the decrease (about 6 times) in the paeoniflorin CL/F value was greater than the decrease (about 1.5–2.5 times) in CL/F of ephedrine and cinnamic acid according to multiple doses of SCRT (compared to a single administration). This would suggest a relatively large inhibition of paeoniflorin metabolism following multiple doses of SCRT. The average V_d_/F values of ephedrine, paeoniflorin, and cinnamic acid decreased in the same manner with multiple doses compared to a single dose of SCRT. This may be related to the degree of plasma protein-binding of the SCRT components. The degrees of plasma protein-binding of ephedrine, paeoniflorin, and cinnamic acid, the main components of SCRT, was about 4–6% [45], 10–20% [46], and 60–65% [18], respectively. Therefore, as SCRT is administered multiple times, competition occurs in plasma protein-binding between the components with the accumulation of components in the blood, and as a result, the V_d_/F values of paeoniflorin and cinnamic acid, which have relatively higher plasma protein-binding degrees than that of ephedrine, may be significantly reduced. In addition, the possibility of various factors such as metabolic inhibition and functional changes in transporters due to multiple SCRT exposures cannot be excluded [47].

The AUC_120–∞_ values obtained after multiple doses were divided by the AUC_0–∞_ values obtained after a single dose to evaluate the level of blood exposure of ephedrine, paeoniflorin, and cinnamic acid according to multiple doses of SCRT. All AUC_120–∞_/AUC_0–∞_ values were larger than 1. Therefore, it could be estimated that the exposure of ephedrine, paeoniflorin, and cinnamic acid in the blood following multiple administrations of SCRT was increased compared to a single administration. In particular, it was found that the degree of increased exposure to paeoniflorin and cinnamic acid was significant. In theory, if the pharmacokinetic properties of each component followed a linear system, then the AUC_120–132_ of multiple doses/AUC_0–∞_ of single-dose values should be nearly 1. However, the reason the average AUC_120–132_ of multiple doses/AUC_0–∞_ of single-dose values of ephedrine, paeoniflorin, and cinnamic acid were 1.11, 5.22, and 2.28, respectively, which is greater than 1, was probably due to the increased blood exposure and the accumulation of ephedrine, paeoniflorin, and cinnamic acid by multiple doses of SCRT. Similarly, theoretically, the C_max_ of multiple doses/C_max_ of single-dose values should be approximately equal to the R values if the pharmacokinetic properties of each component followed a linear system. The average C_max_ of multiple doses/C_max_ of single-dose values of ephedrine, paeoniflorin, and cinnamic acid calculated from this study were 1.03, 9.96, and 4.07, respectively. In the case of ephedrine, the calculated C_max_ of multiple doses/C_max_ of a single dose was almost the same as the R-value (Table 2). However, in the case of paeoniflorin and cinnamic acid, the C_max_ of multiple doses/C_max_ of a single dose was greater than the estimated R values (Table 2). The reason was probably that, as mentioned above, the exposure and accumulation of paeoniflorin and cinnamic acid in the blood compared to ephedrine were significantly increased beyond the linear range following multiple doses of SCRT.

Like other traditional herbal medicines made from an average of more than 7–10 kinds of herbs, SCRT tablets contain a mixture of eight different herbs [7]. Therefore, the herbal medicines administered in multiple doses can exhibit different pharmacokinetic parameters in the body compared to single-dose administrations due to the interaction between the components or the effect of certain components contained in the formulation on the gastrointestinal tract [13,14]. For example, ephedrine, a marker component of *Ephedrae Herba*, is known to suppress gastric acid secretion and gastrointestinal motility [48,49]; and cinnamic acid, an indicator component of *Cinnamoni Ramulus*, has been reported to inhibit gastrointestinal motility and spasms in mice [50]. Therefore, multiple doses of SCRT may increase the absorption of components such as paeoniflorin and cinnamic acid into the blood by inhibiting excretion through the gastrointestinal tract. To clarify these findings, further research on the effects of SCRT on the gastrointestinal tract is needed.

The standard deviation of the overall pharmacokinetic parameter values (including AUC, t_1/2_, C_max_, T_max_, CL/F, and V_d_/F) of cinnamic acid according to a single or multiple oral administration of SCRT was relatively larger than those of ephedrine and paeoniflorin (shown in Table 2 and Table 3). This suggested that there was a large difference between individuals in the pharmacokinetics of cinnamic acid following the oral administration of SCRT. Therefore, in relation to the pharmacokinetic diversity among individuals of cinnamic acid, it is necessary to explore significant covariates and population pharmacokinetic analysis approaches that can explain the pharmacokinetic diversity (of cinnamic acid) in the future.

In this study, we focused on providing a new perspective on the nonlinear pharmacokinetic system of herbal medicines. Among the herbal medicines, SCRT tablets, which are registered to health insurance drugs in Korea and used frequently, were selected as an example. In this study, we ultimately confirmed that the pharmacokinetic parameters of SCRT were changed according to single and multiple doses. That is, it was confirmed that the components of ephedrine, paeoniflorin, and cinnamic acid set as targets within SCRT were significantly accumulated in the plasma according to the multiple administrations of SCRT. We believe that the changes in pharmacokinetic profiles according to multiple doses of herbal medicines should be considered very important in future studies on the toxicity and efficacy of herbal medicines. Until now, studies on the pharmacokinetics of herbal medicines have mainly focused on the presentation of the profile of the ingredients according to a single administration. The results we present in this study indicate that we expect that the changes in the pharmacokinetics of the active ingredients according to multiple administrations of herbal medicines will be an important reference for setting the dosage and administration of herbal medicines in clinical applications.

## 4. Conclusions

Relatively simple and sensitive UHPLC–MS/MS methods were applied and well-validated to determine the ephedrine, paeoniflorin, and cinnamic acid concentrations in rat plasma. The LLOQs of ephedrine, paeoniflorin, and cinnamic acid achieved 0.5, 0.2, and 0.1 ng/mL, respectively, which were sufficiently low concentrations to study the pharmacokinetic profiles in rats. This study was the first to evaluate the pharmacokinetics of ephedrine, paeoniflorin, and cinnamic acid after single or multiple oral doses of SCRT to rats. In multiple doses of SCRT, the main constituents, ephedrine, paeoniflorin, and cinnamic acid, were found to increase blood exposure compared to a single dose. The results of this study are expected to provide useful pharmacokinetic data for the safety and efficacy evaluations of SCRT in the future. This study also presented a typical example of data showing pharmacokinetic changes according to the repeated administration of an herbal medicine compared to a single administration. Therefore, based on the results of this study, it is necessary to conduct studies on the relationship between the pharmacokinetic variability (according to multiple doses of herbal medicines) and clinical efficacy in the future. In addition, following this study, advanced nonlinear pharmacokinetic studies such as dose-escalation studies that can explain the nonlinear pharmacokinetic characteristics of major components of SCRT, paeoniflorin and cinnamic acid, and the presentation of the inter-individual variability through population pharmacokinetic analysis, will need to be conducted. In this regard, the potential studies on alterations in the pharmacokinetics according to the interaction among components, gender, age, and diet, and a population pharmacokinetic analysis study that considers these as covariates may be examples.

## Figures and Tables

**Table 1:** Optimized mass spectrometry analysis conditions of analytes.

Compound	Polarity	Parent ion (*m*/*z*)	Product ion (*m*/*z*)	Retention Time (min)	Q1 Pre-Bias (V)	Q3 Pre-Bias (V)	Collision Energy (eV)
Ephedrine	Positive	166.20	148.10	2.37	22	27	12
Paeoniflorin	Negative	525.20	449.25	3.32	−26	−24	−15
Cinnamic acid	Negative	146.80	103.00	2.42	−13	−24	−13
Diphenhydramine (IS)	Positive	256.10	167.05	2.57	18	11	19
Geniposide (IS)	Negative	432.90	225.00	2.88 (for paeoniflorin) 2.17 (for cinnamic acid)	−16	−26	−14

IS refers to internal standard.

**Table 2:** Main pharmacokinetic parameters for the three components in rats after a single oral administration of SCRT containing 0.49 mg/kg of ephedrine, 2.475 mg/kg of paeoniflorin, and 0.0125 mg/kg of cinnamic acid. All values are presented as the mean ± standard deviation (SD).

Parameters		Ephedrine	Paeoniflorin	Cinnamic Acid
		Oral (*n* = 5)	Oral (*n* = 5)	Oral (*n* = 5)
AUC_0-12_	ng·h/mL	54.76 ± 15.09	65.72 ± 23.25	192.00 ± 59.56
AUC_0-∞_	ng·h/mL	57.67 ± 17.68	66.33 ± 23.34	322.27 ± 182.53
t_1/2_	h	1.62 ± 0.74	1.27 ± 0.55	9.30 ± 8.31
C_max_	ng/mL	20.39 ± 7.83	23.54 ± 10.01	67.42 ± 23.23
T_max_	h	0.85 ± 0.22	1.40 ± 0.55	0.35 ± 0.14
CL/F	L/h/kg	9.19 ± 2.97	42.52 ± 19.52	0.05 ± 0.03
V_d_/F	L/kg	20.50 ± 8.22	74.05 ± 33.52	0.49 ± 0.20
R ^†^	-	1.03 ± 0.01	1.01 ± 0.01	2.23 ± 0.00

^†^ R is the accumulation ratio calculated by the following equation: 1 / (1−e ^−k·τ^), where k is the elimination rate constant and τ is the dosing interval (8 h).

**Table 3:** Main pharmacokinetic parameters for the three components in rats after multiple oral administrations of SCRT containing 0.49 mg/kg of ephedrine, 2.475 mg/kg of paeoniflorin, and 0.0125 mg/kg of cinnamic acid. All data are presented as the mean ± SD.

Parameters		Ephedrine	Paeoniflorin	Cinnamic Acid
		Oral (*n* = 5)	Oral (*n* = 5)	Oral (*n* = 5)
AUC_120–132_	ng·h/mL	63.84 ± 27.45	346.34 ± 81.42 *	734.64 ± 435.73 *
AUC_120-∞_	ng·h/mL	77.32 ± 13.98	356.97 ± 89.80 *	1615.84 ± 1904.37
t_1/2_	h	2.01 ± 0.35	2.33 ± 0.12 *	11.47 ± 10.54
C_max_	ng/mL	21.06 ± 5.80	234.46 ± 57.51 *	274.66 ± 122.79 *
T_max_	h	0.85 ± 0.22	0.60 ± 0.14 *	0.25 ± 0.00 *
CL/F	L/h/kg	6.49 ± 1.14	7.32 ± 1.92 *	0.02 ± 0.01 *
V_d_/F	L/kg	18.66 ± 3.76	24.42 ± 5.48 *	0.20 ± 0.11 *
AUC_120-∞_ / AUC_0-∞_^†^	-	1.34	5.38	5.01

^†^ AUC_120-∞_ and AUC_0-∞_ are the mean AUC values of SCRT multiple or single doses, respectively. * *p* < 0.05 between the single and multiple administration groups.

## Data Availability

The data presented in this study are available in the article and Supplementary Materials.

## Supplementary Materials

The following are available online at https://www.mdpi.com/1999-4923/13/4/478/s1, Table S1. Summary of UHPLC-MS/MS method validation items; Table S2. Summary of test results under several conditions performed for the optimal separation of ephedrine, paeoniflorin, and cinnamic acid; Table S3. Precision and accuracy of UHPLC-MS/MS analysis for the determination of ephedrine, paeoniflorin, and cinnamic acid in rat plasma (mean ± SD, *n* = 5); Table S4. Recovery and matrix effect for the determination of ephedrine, paeoniflorin, and cinnamic acid in rat plasma (mean ± SD, *n* = 5); Table S5. Stability (%) of ephedrine, paeoniflorin, and cinnamic acid in rat plasma under various conditions (mean ± SD, *n* = 5); Table S6. Stability of stock and working solutions of ephedrine, paeoniflorin, and cinnamic acid at −20 °C for eight weeks (mean ± SD, *n* = 5); Table S7. Summary of previously reported pharmacokinetic parameter results for ephedrine, paeoniflorin, and cinnamic acid after the single oral administration of various herbal medicines or internal standards; Figure S1. A schematic diagram summarizing the experimental design; Figure S2. Precursor and product ion mass spectra of ephedrine (A), paeoniflorin (B), cinnamic acid (C), diphenhydramine (D), and geniposide (E) in the positive and negative ionization modes; Figure S3. Representative MRM chromatograms of ephedrine (A), paeoniflorin (B), and cinnamic acid (C) with the IS; Figure S4. MRM chromatograms of ephedrine in blank plasma (A), zero plasma containing the IS (B), and a plasma sample at 0.75 h after the oral administration of a SCRT tablet (C); Figure S5. MRM chromatograms of paeoniflorin in blank plasma (A), zero plasma containing the IS (B), and a plasma sample at 0.75 h after the oral administration of an SCRT tablet (C); Figure S6. MRM chromatograms of cinnamic acid in blank plasma (A), zero plasma containing the IS (B), and a plasma sample at 0.75 h after the oral administration of an SCRT tablet (C).

## Abbreviations

SCRT	Socheongryong-tang
UHPLC-MS/MS	Ultrahigh-performance liquid chromatography-tandem mass spectrometer
HPLC	High-performance liquid chromatography
ESI	Electrospray ionization
MRM	Multiple reaction monitoring
LLOQ	Lower limit of quantitation
IS	Internal standard
QC	Quality control
CV	Coefficient of variation
SD	Standard deviation
