# Supplementary material for: Pharmacokinetic Changes According to Single or Multiple Oral Administrations of Socheongryong-Tang to Rats: Presented as a Typical Example of Changes in the Pharmacokinetics Following Multiple Exposures to Herbal Medicines
Source: Pharmaceutics. 2021 Apr 1;13(4):478. doi: 10.3390/pharmaceutics13040478 (PMC8103508; doi:10.3390/pharmaceutics13040478)
Supplement: Supplementary file 1 [file pharmaceutics-13-00478-s001.zip › pharmaceutics-1159914-SI.pdf]

pharmaceutics

Supplementary Materials: Pharmacokinetic Changes According to Single or Multiple Oral Administrations of Socheongryong-tang to rats: Presented as a Typical Example of Changes in the Pharmacokinetics Following Multiple Exposures to Herbal Medicines

Seung-Hyun Jeong, Ji-Hun Jang, Da-Hwa Jung, Guk-Yeo Lee and Yong-Bok Lee

Citation: Jeong, S.-H.; Jang, J.-H.; Jung, D.-H.; Lee, G.-Y.; Lee, Y.-B. Pharmacokinetic Changes According to Single or Multiple Oral Administrations of Socheongryong-tang to rats: Presented as a Typical Example of Changes in the Pharmacokinetics Following Multiple Exposures to Herbal Medicines. *Pharmaceutics* **2021**, *13*, 487. https://doi.org/10.3390/pharmaceutics13040478

Academic Editor: Kishor M. Wasan

Received: 11 March 2021
Accepted: 29 March 2021
Published: 1 April 2021

Publisher's Note: MDPI stays neutral with regard to jurisdictional claims in published maps and institutional affiliations.

Copyright: © 2021 by the authors. Submitted for possible open access publication under the terms and conditions of the Creative Commons Attribution (CC BY) license (http://creativecommons.org/licenses/by/4.0/).

Table S1. Summary of UHPLC-MS/MS method validation items.

Validation item	Description
Selectivity and sensitivity	Selectivity was investigated to confirm the influence of endogenous compounds located in the closed retention times for the analytes. Blank plasma from healthy and drug-free rats; $n \geq 6$; plasma spiked with the IS (as zero); and plasma samples obtained after the oral administration of 120 mg/kg of Socheongryong-tang (SCRT) to rats were used to determine selectivity. The sensitivity of the method was expressed as the lower limit of quantitation (LLOQ) determined as the lowest concentration of the standard samples with a signal-to-noise ratio of at least 10:1, in accordance with an acceptable precision of less than 20% and an accuracy within ± 20%, which were evaluated using five replicate samples.
Linearity	Calibration curves were constructed by linear regression using 6 –8 calibration points with a weighting factor of 1/concentration^2. Linearity was determined by plotting the analyte/IS peak area versus the theoretical analyte concentration, suggesting a linear calibration equation with its correlation coefficient (r^2). The linearity of ephedrine, paeoniflorin, and cinnamic acid was estimated using a series of calibration standards in the range of 0.5 – ,000 ng/mL, 0.2 – 20 ng/mL, and 0.1 – 500 ng/mL, respectively, in rat plasma. A straight-line regression equation was obtained with an r^2 value of 0.99 or more.
Precision and accuracy	Intra-batch precision and accuracy were determined by analyzing the QC samples (LLOQ = 0.5, 0.2, and 0.1 ng/mL for ephedrine, paeoniflorin, and cinnamic acid, respectively; low concentrations = 1, 0.6, and 0.3 ng/mL for ephedrine, paeoniflorin, and cinnamic acid, respectively; medium concentrations = 500, 10, and 250 ng/mL for ephedrine, paeoniflorin, and cinnamic acid; and high concentrations = 800, 16, and 400 ng/mL for ephedrine, paeoniflorin, and cinnamic acid, respectively) at five different times on the same day. Inter-batch assessments were similarly carried out on five consecutive days. The concentration of each QC sample was evaluated using freshly prepared calibration standards, and the precision was determined by calculating the coefficient of variation (CV) in the analysis of the QC samples. The precision CV for each concentration level should not deviate by more than ± 15% except for the LLOQ with a limit of 20%. The accuracy was evaluated based on the criterion of a mean no greater than 15% of the nominal concentration except for the LLOQ, which should not exceed 20%.
Recovery and matrix effect	The recovery of ephedrine, paeoniflorin, and cinnamic acid were evaluated for the QC samples at low, medium, and high concentrations in five replicates. The extraction recoveries for the three analytes from rat plasma were assessed by comparing the detector (MS/MS) response for the extracted samples (A) to those of the samples added at the same concentration after extracting the blank plasma (B). Recovery of the ISs was evaluated at working concentrations of 10 ng/mL for geniposide and 4 ng/mL for diphenhydramine in the same manner. Additionally, the matrix effect was evaluated by comparing the peak area of the analyte post-extraction (B) in blank plasma with the absolute standard (C) of the same. The recovery and matrix effect were calculated as follows: Recovery = $\frac{A}{B} \times 100\%$; Matrix effect = $\frac{B}{C} \times 100\%$. The recovery did not need to be 100%, but the value should have been consistent, precise, and reproducible. Additionally, a matrix effect of 100% indicated that the matrix components had little effect on the quantification of ephedrine, paeoniflorin, and cinnamic acid.
Stability	Studies were designed to evaluate the stability of ephedrine, paeoniflorin, and cinnamic acid in rat plasma samples under various storage and process conditions of short-term and long-term storage, freeze-thaw, and autosampler (post-preparative) conditions. Two concentrations of QC samples were examined in all stability tests: low: 1, 0.6, and 0.3 ng/mL for ephedrine, paeoniflorin, and cinnamic acid, respectively; and high: 800, 16, and 400 ng/mL for ephedrine, paeoniflorin, and cinnamic acid, respectively. Short-term stability was tested by maintaining the QC samples at room temperature (25 °C) for 4 or 24 h, and long-term stability was measured by analyzing QC samples that were frozen at -80 °C for 1 – 8 weeks. For the freeze-and-thaw stability test, the QC samples were stored at -80 °C for 24 h and then thawed completely at 25°C. This cycle was repeated, and the analysis was performed after the first or third cycle. In addition, the QC samples were placed in the autosampler at 15 °C for 24 h to test the post-preparative stability. The stability of the stock

	solutions of ephedrine, paeoniflorin, cinnamic acid, and IS was assessed by measuring the analyte concentrations after storage at -20 °C for eight weeks. The samples were considered stable if the mean peak area at each level was within ± 15% of the sample nominal concentration and the precision was less than 15% (*n* = 5).
Carryover	Carryover was tested to determine whether the analytes or the IS remaining in the analytical instrument would affect the analysis and quantification of subsequent sample measurements. The carryover was tested by injecting a blank sample after injecting the maximum concentration sample of each analyte (1,000, 20, and 500 ng/mL for ephedrine, paeoniflorin, and cinnamic acid; 10 and 20 ng/mL for IS). In this blank sample, each analyte peak should have been less than 20% of the LLOQ peak.
Dilution integrity	Dilution integrity was tested to confirm that dilutions (when the concentration of the sample exceeded the maximum quantitative limit of 1,000, 20, or 500 ng/mL in ephedrine, paeoniflorin, and cinnamic acid, respectively) made by adding the same biological matrix did not affect the analysis. Specifically, a sample that exceeded 1,000 or 20 or 500 ng/mL in ephedrine, paeoniflorin, and cinnamic acid was diluted with a biological matrix and analyzed five times for each dilution factor, and whether the concentration of the diluted sample was within the calibration curve range was determined.

Table S2. Summary of test results under several conditions performed for the optimal separation of ephedrine, paeoniflorin, and cinnamic acid.

Compound	Mobile phase test	Column test	Sample preparation test
Ephedrine	The addition of 0.1% (*v*/*v*) formic acid to mobile phase A increased peak intensity compared to 0.05% (*v*/*v*) formic acid. When 100% methanol was used as mobile phase B, the peak sensitivity of the analyte was increased and the noise was reduced compared to 100% acetonitrile.	Peak cleavage was not observed when analyzing ephedrine with the HALO-C_{18} column. There was no overlap of the IS and analyte peak retention times.	For ephedrine, the detection sensitivity was excellent in this condition and sample preparation (using methanol) was possible without decompression with centrifugal evaporation using nitrogen.
Paeoniflorin	The addition of 0.1% (*v*/*v*) formic acid to mobile phase A resulted in increased peak intensity compared to 0.05% (*v*/*v*) formic acid. Using 100% acetonitrile as mobile phase B increased the peak sensitivity and elution of the analyte.	The sensitivity of paeoniflorin using the Phenomenex Kinetex core-shell biphenyl column was very high. Peak symmetry was also excellent and there was no tailing phenomenon.	The methanol-based protein precipitation method showed less noise and superior sensitivity compared to the liquid-liquid extraction (using ethyl-acetate or ether) method. The effect was better using methanol compared to acetonitrile as the protein precipitation method.
Cinnamic acid	The mobile phase condition of acetonitrile and water containing 2 mM ammonium acetate or 100% water was attempted. However, these results were unsatisfactory for cinnamic acid analysis due to unsuitable resolution and low sensitivity. Formic acid in water (0.005% (*v*/*v*)) as mobile phase A and acetonitrile as mobile phase B displayed the highest intensity and best resolution.	HALO-C_{18}, Inertsil-C_8, UPLC® BEH C_{18}, and Phenomenex Kinetex core-shell biphenyl columns were tested to obtain an optimum chromatogram. The HALO-C_{18} column was more suitable than the others for analyzing cinnamic acid. Peak sensitivity and symmetry were excellent.	For cinnamic acid, ethyl acetate extracted the largest amount compared to methyl-t-butyl ether, methylene chloride, and di-ethyl ether. Acetic acid was added to the extraction solvents to suppress the ionization of cinnamic acid and then to increase the transfer of cinnamic acid to the organic solvent layer. The best extraction efficiency was obtained when a mixed organic solvent of methanol and ethyl acetate with added acetic acid was used as the extraction solvent.

Table S3. Precision and accuracy of UHPLC-MS/MS analysis for the determination of ephedrine, paeoniflorin, and cinnamic acid in rat plasma (mean ± SD, *n* = 5).

Spiked Conc. (ng/mL)	Intra-Batch (*n* = 5)			Inter-batch (*n* = 5)		
	Measured Conc. (ng/mL, mean ± SD)	Precision (CV, %)	Accuracy (%)	Measured Conc. (ng/mL, mean ± SD)	Precision (CV, %)	Accuracy (%)
			**Ephedrine**			
0.5	0.49 ± 0.01	2.27	98.77	0.51 ± 0.02	5.56	103.22
1	1.04 ± 0.02	1.66	102.11	1.02 ± 0.03	2.86	101.43
500	487.50 ± 14.69	2.90	96.13	487.59 ± 11.25	2.20	95.81
800	747.03 ± 10.10	1.25	92.00	739.00 ± 14.51	2.40	90.81
			**Paeoniflorin**			
0.2	0.23 ± 0.021	9.19	110.87	0.21 ± 0.010	5.04	101.60
0.6	0.66 ± 0.030	5.20	104.67	0.58 ± 0.052	8.21	94.57
10	11.22 ± 0.43	3.67	106.87	10.17 ± 0.56	5.49	99.93
16	17.43 ± 1.541	8.83	103.77	16.04 ± 0.844	5.26	98.63
			**Cinnamic acid**			
0.1	0.10 ± 0.00	1.96	102.00	0.10 ± 0.00	3.88	98.75
0.3	0.30 ± 0.01	4.13	101.70	0.29 ± 0.01	3.64	95.60
250	240.16 ± 6.69	2.57	96.07	266.72 ± 7.75	2.90	106.69
400	425.40 ± 9.43	2.22	106.35	414.63 ± 11.38	2.75	103.66

Table S4. Recovery and matrix effect for the determination of ephedrine, paeoniflorin, and cinnamic acid in rat plasma (mean ± SD, *n* = 5).

Spiked Conc. (ng/mL)	Recovery (%)	Matrix effect (%)
	**Ephedrine**	
1	73.93 ± 4.27	98.26 ± 1.77
500	74.28 ± 4.85	100.24 ± 1.80
800	76.49 ± 4.11	95.96 ± 3.46
	**Paeoniflorin**	
0.6	82.45 ± 5.88	100.44 ± 0.91
10	84.29 ± 4.82	97.40 ± 3.23
16	84.81 ± 5.21	99.93 ± 2.05
	**Cinnamic acid**	
0.3	79.34 ± 3.71	99.08 ± 1.32
250	77.18 ± 5.39	98.77 ± 3.06
400	81.46 ± 4.88	101.29 ± 2.02

Table S5. Stability (%) of ephedrine, paeoniflorin, and cinnamic acid in rat plasma under various conditions (mean ± SD, *n* = 5).

Spiked Conc. (ng/mL)	Short-term[1] (4 h, 25 °C)	Short-term[2] (24 h, 25 °C)	Long-term[1] (1 week, −80 °C)	Long-term[2] (4 weeks, −80 °C)	Long-term[3] (8 weeks, −80 °C)	Autosampler (24 h, 15 °C)	Freeze-thaw[1] (1 cycle, from −80 °C to 25 °C)	Freeze-thaw[2] (3 cycles, from −80 °C to 25 °C)
**Ephedrine**								
1	98.46 ± 4.32	95.63 ± 3.03	96.36 ± 3.02	96.43 ± 5.32	99.30 ± 1.48	96.34 ± 3.53	93.33 ± 4.62	97.36 ± 2.53
800	97.01 ± 6.81	98.35 ± 1.55	98.45 ± 4.35	94.58 ± 6.12	95.37 ± 2.12	98.01 ± 4.99	96.21 ± 3.34	102.00 ± 6.92
**Paeoniflorin**								
0.6	95.54 ± 4.85	97.46 ± 3.73	93.68 ± 4.98	94.06 ± 5.51	95.66 ± 3.33	96.47 ± 3.57	97.84 ± 2.17	100.26 ± 3.53
16	96.83 ± 1.99	95.97 ± 2.91	98.74 ± 2.04	99.63 ± 3.10	93.72 ± 6.91	96.81 ± 3.31	99.54 ± 1.93	102.52 ± 6.82
**Cinnamic acid**								
0.3	100.35 ± 2.12	99.18 ± 2.05	99.87 ± 1.58	101.76 ± 2.33	97.98 ± 3.15	98.59 ± 2.25	100.23 ± 1.96	98.89 ± 2.55
400	99.43 ± 2.40	100.56 ± 2.19	100.21 ± 1.99	98.34 ± 2.51	102.01 ± 2.96	98.80 ± 2.68	99.70 ± 2.51	100.36 ± 2.84

Table S6. Stability of stock and working solutions of ephedrine, paeoniflorin, and cinnamic acid at −20 °C for eight weeks (mean ± SD, *n* = 5).

Compound	Stock Solution		Working Solution	
	Concentration (mg/mL)	Stability (%)	Concentration (ng/mL)	Stability (%)
Ephedrine	1.00	100.28 ± 4.32	5	99.13 ± 1.44
			10,000	96.41 ± 3.59
Paeoniflorin	1.00	99.44 ± 2.47	2	100.17 ± 1.16
			200	98.26 ± 2.48
Cinnamic acid	1.00	97.18 ± 2.92	1	97.49 ± 2.83
			5,000	98.45 ± 3.47
Diphenhydramine (IS)	1.00	98.52 ± 4.06	100	97.15 ± 4.24
Geniposide (IS)	1.00	100.39 ± 2.48	100	98.38 ± 2.26

IS meant internal standard.

Table S7. Summary of previously reported pharmacokinetic parameter results for ephedrine, paeoniflorin, and cinnamic acid after the single oral administration of various herbal medicines or internal standards.

Analytes	T_{max} (h)	$t_{1/2}$ (h)	C_{max} (ng/mL)	Dose (mg/kg)	Administration Route	Dosage Form	Reference
Ephedrine	1.0	2.59 ± 0.79	1661.92 ± 86.23	-[a]	Oral	*Mahuang* decoction	Wan et al., 2020 [1]
	1.0	1.68 ± 0.59	851.53 ± 40.74	-[a]	Oral	*Mahuang* decoction	Wan et al., 2020 [1]
	1.0	1.93 ± 0.33	485.80 ± 35.22	-[a]	Oral	*Mahuang* decoction	Wan et al., 2020 [2]
	0.75	2.17 ± 0.36	4150 ± 670	20	Oral	*Ephedra* decoction	Tang et al., 2017 [2]
	1.75 ± 0.45	4.12 ± 0.96	417 ± 51.1	31.1	Oral	Maxingshiga-tang	Wang et al., 2016 [3]
	3.08 ± 0.61	2.22 ± 0.28	383 ± 36.8	31.1	Oral	*Ephedra* extract	Wang et al., 2016 [3]
	0.29 ± 0.04	1.57 ± 0.13	1290 ± 172	31.1	Oral	Standard ephedrine	Wang et al., 2016 [3]
	1.67 ± 0.58	1.15 ± 0.32	46.85 ± 18.79	1.24	Oral	*Keke* capsule originating from Maxingshiga-tang	Song et al., 2014 [4]
	0.33 ± 0.20	9.76 ± 5.56	1180.44 ± 329.50	-[a]	Oral	*Mahuang* aqueous extracts	Wei et al., 2014 [5]
	0.92-1.33	1.00-2.49	1547.55-2556.87	-[a]	Oral	*Mahuang-Guizhi herb-pair* aqueous extracts	Wei et al., 2014 [5]
Paeoniflorin	2.70 ± 0.27	1.45 ± 0.22	340 ± 50	100	Oral	Standard paeoniflorin	Wang et al., 2016 [6]
	0.37 ± 0.13	5.65 ± 1.06	5686.12 ± 1496.20	119.8	Oral	*Cerebralcare* granule	Wang et al., 2013 [7]
	0.36-0.44	4.51-5.26	743.83-12830	-[a]	Oral	*Shaoyao-Gancao* decoction	Xu et al., 2013 [8]
	0.08 ± 0.00	4.24 ± 0.88	7350 ± 2980	7000	Oral	*Radix Paeoniae Rubra* decoction	Jiang et al., 2012 [9]
	0.44 ± 0.21	4.51 ± 1.04	8010 ± 2190	14000	Oral	*Radix Paeoniae Rubra* decoction	Jiang et al., 2012 [9]

	0.53–0.74	1.87-2.21	360–470	80	Oral	Samul-tang	Hwang et al., 2012 [10]
	0.5	2.22 ± 0.39	1550 ± 12	182.7	Oral	*Paeoniae Radix* decoction	Gan et al., 2012 [11]
	0.5	2.32 ± 0.40	1414 ± 9	165.7	Oral	Shaoyao-Gancao-tang	Gan et al., 2012 [11]
	0.50 ± 0.00	6.94 ± 1.22	2240 ± 310	224.4	Oral	Standard paeoniflorin	Liu et al., 2011 [12]
	0.37 ± 0.08	5.95 ± 1.53	5150 ± 2100	224.4	Oral	Danggui-Shaoyao-San	Liu et al., 2011 [12]
	0.67 ± 0.07	1.86 ± 0.27	185.24 ± 26.24	-[a]	Oral	*Radix Paeoniae Rubra* decoction	Feng et al., 2010 [13]
	0.33 ± 0.02	0.85 ± 0.11	34.44 ± 13.42	-[a]	Oral	*Radix Paeoniae Alba* decoction	Feng et al., 2010 [13]
	0.75	1.19 ± 0.33	570 ± 50	30	Oral	Standard paeoniflorin	Wu et al., 2009 [14]
	0.30 ± 0.11	1.67 ± 0.35	410 ± 50	30	Oral	*Cortex Moutan* extract	Wu et al., 2009 [14]
	2.50	1.78 ± 0.32	380 ± 90	30	Oral	*Shuang-Dan* decoction	Wu et al., 2009 [14]
	0.58 ± 0.34	4.27 ± 1.57	3340 ± 1180	300	Oral	Standard paeoniflorin	Wang et al., 2008 [15]
	1.67 ± 0.43	6.19 ± 2.06	3690 ± 1460	300	Oral	*Radix Paeoniae Rubra* decoction	Wang et al., 2008 [15]
	0.80 ± 0.35	3.58 ± 0.61	1460 ± 290	300	Oral	*Radix Paeoniae Alba* decoction	Wang et al., 2008 [15]
	0.75 ± 0.08	0.92 ± 0.42	1260 ± 230	150	Oral	Standard paeoniflorin	Liu et al., 2005 [16]
	0.15 ± 0.01	0.83 ± 0.17	9.8 ± 2.1	0.5	Oral	Standard paeoniflorin	Takeda et al., 1995 [17]
	0.16 ± 0.04	1.34 ± 0.39	30.7 ± 2.4	2.0	Oral	Standard paeoniflorin	Takeda et al., 1995 [17]
	0.17 ± 0.04	0.52 ± 0.08	101.5 ± 18.6	5.0	Oral	Standard paeoniflorin	Takeda et al., 1995 [17]
Cinnamic acid	1.0	1.90-3.24	342.51–448.44	-[a]	Oral	*Mahuang* decoction	Wan et al., 2020 [1]

0.08 ± 0.00	2.86 ± 0.72	664.1 ± 172.4	-[a]	Oral	Huangqi-Guizhi-Wuwu decoction	Guan et al., 2019 [18]
0.083 ± 0.05	2.45 ± 0.46	36.20 ± 3.52	-[a]	Oral	Ling-Gui-Zhu-Gan decoction	Ji et al., 2018 [19]
0.3 ± 0.2	1.0 ± 0.5	5790 ± 246	-[a]	Oral	*Cinnamoni Ramulus* extract	Ji et al., 2015 [20]
0.13 ± 0.05	1.69 ± 0.17	1041.8 ± 247.8	7.2	Oral	Guizhi-Fuling Capsule	Zhao et al., 2015 [21]
0.5 ± 0.00	4.14 ± 0.25	1021.32 ± 90.55	10	Oral	Standard cinnamic acid	Basu et al., 2013 [22]
1.48 ± 0.14	2.5 ± 0.9	556.8 ± 94.2	37.2	Oral	Xuanshen (*Radix Scrophulariae*) extract	Li et al., 2007 [23]
0.12 ± 0.1	0.33 ± 0.1	20742 ± 14816	11.29	Oral	*Cinnamoni Ramulus* decoction	Chen et al., 2009 [24]

[a] means that the exact dosage for each ingredient was not presented in the reports. Only information on herbal medicinal herbs was presented, and accurate content information and dosages administered to the rats for each component were limited.

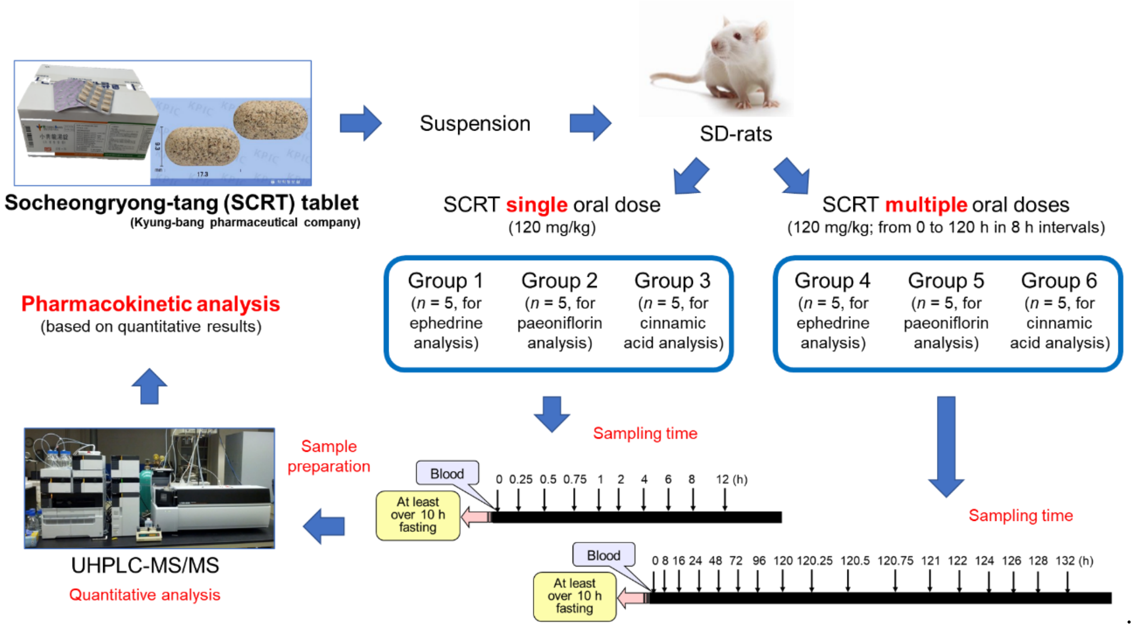

Figure S1. A schematic diagram summarizing the experimental design.

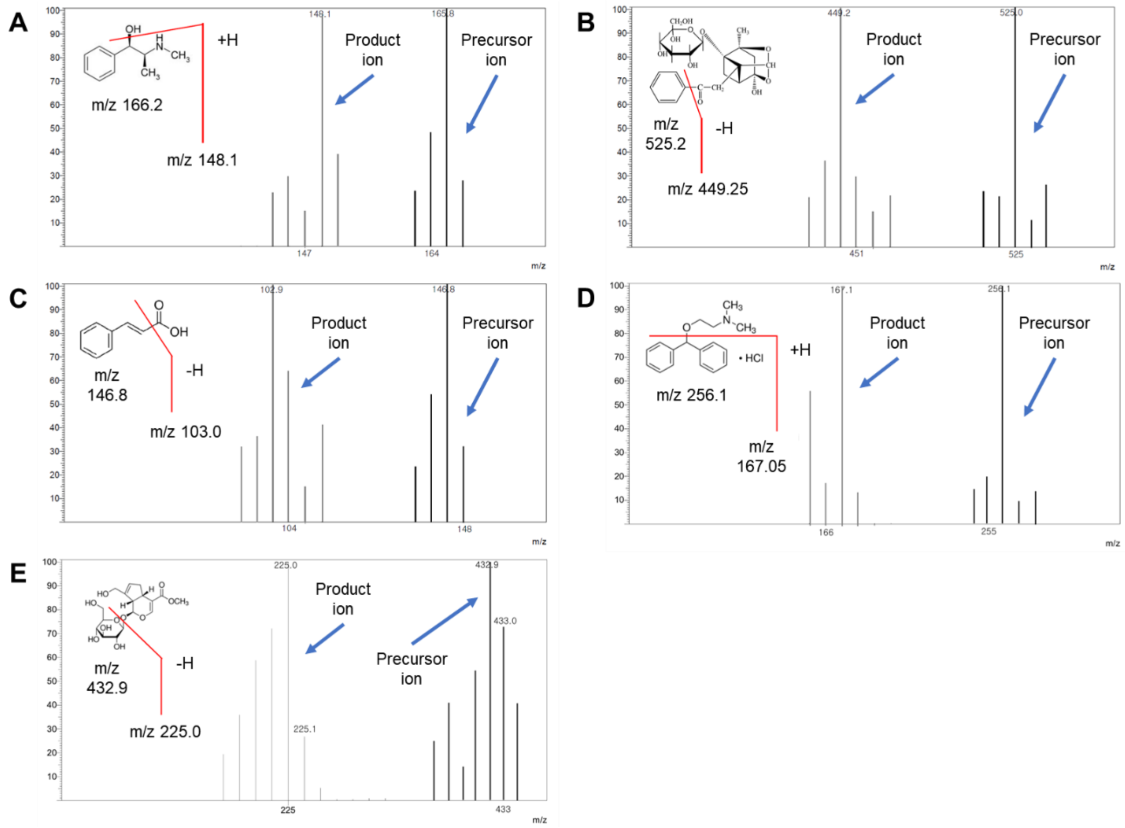

Figure S2. Precursor and product ion mass spectra of ephedrine (**A**), paeoniflorin (**B**), cinnamic acid (**C**), diphenhydramine (**D**), and geniposide (**E**) in the positive and negative ionization modes.

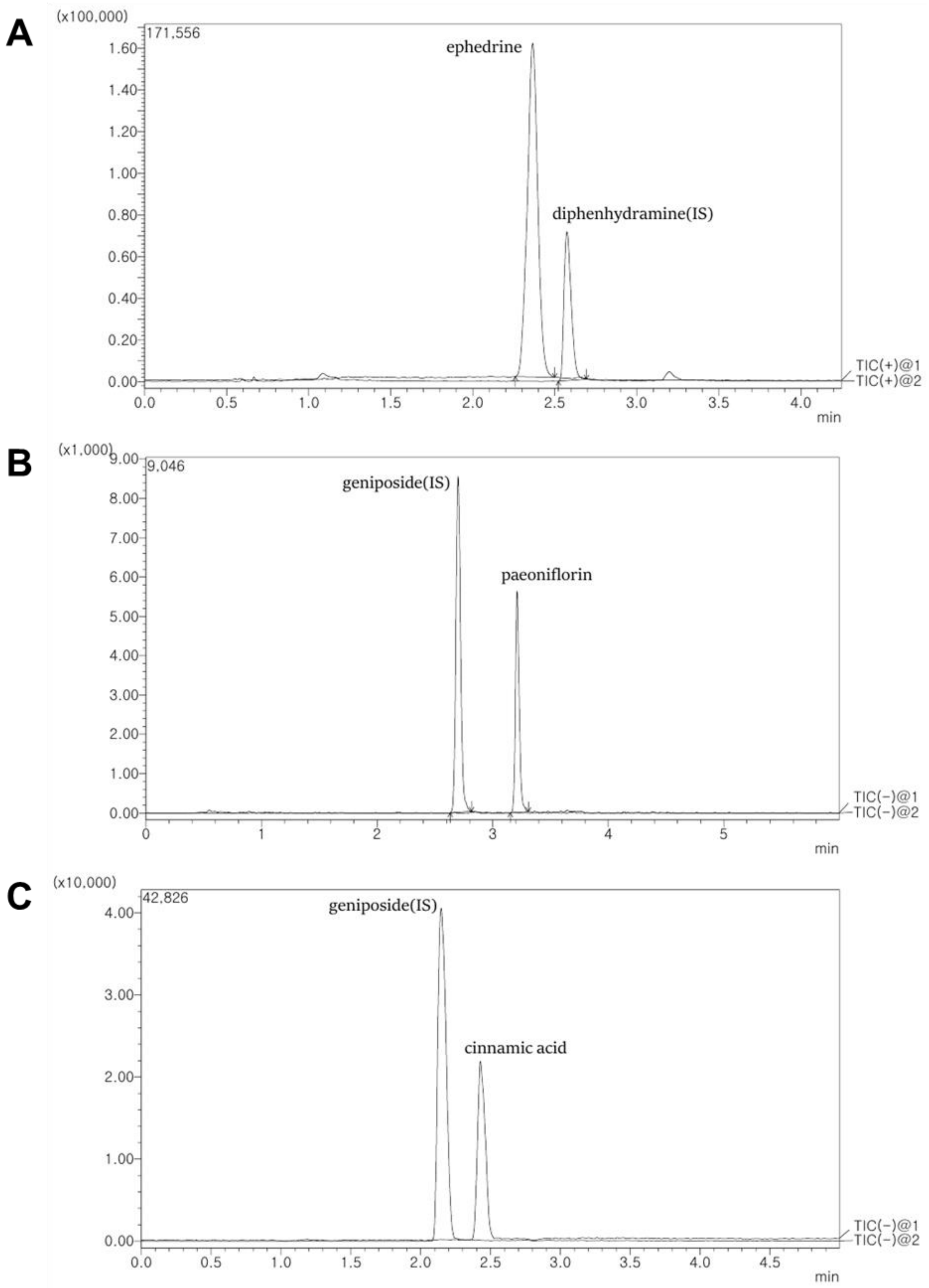

Figure S3. Representative MRM chromatograms of ephedrine (**A**), paeoniflorin (**B**), and cinnamic acid (**C**) with the IS.

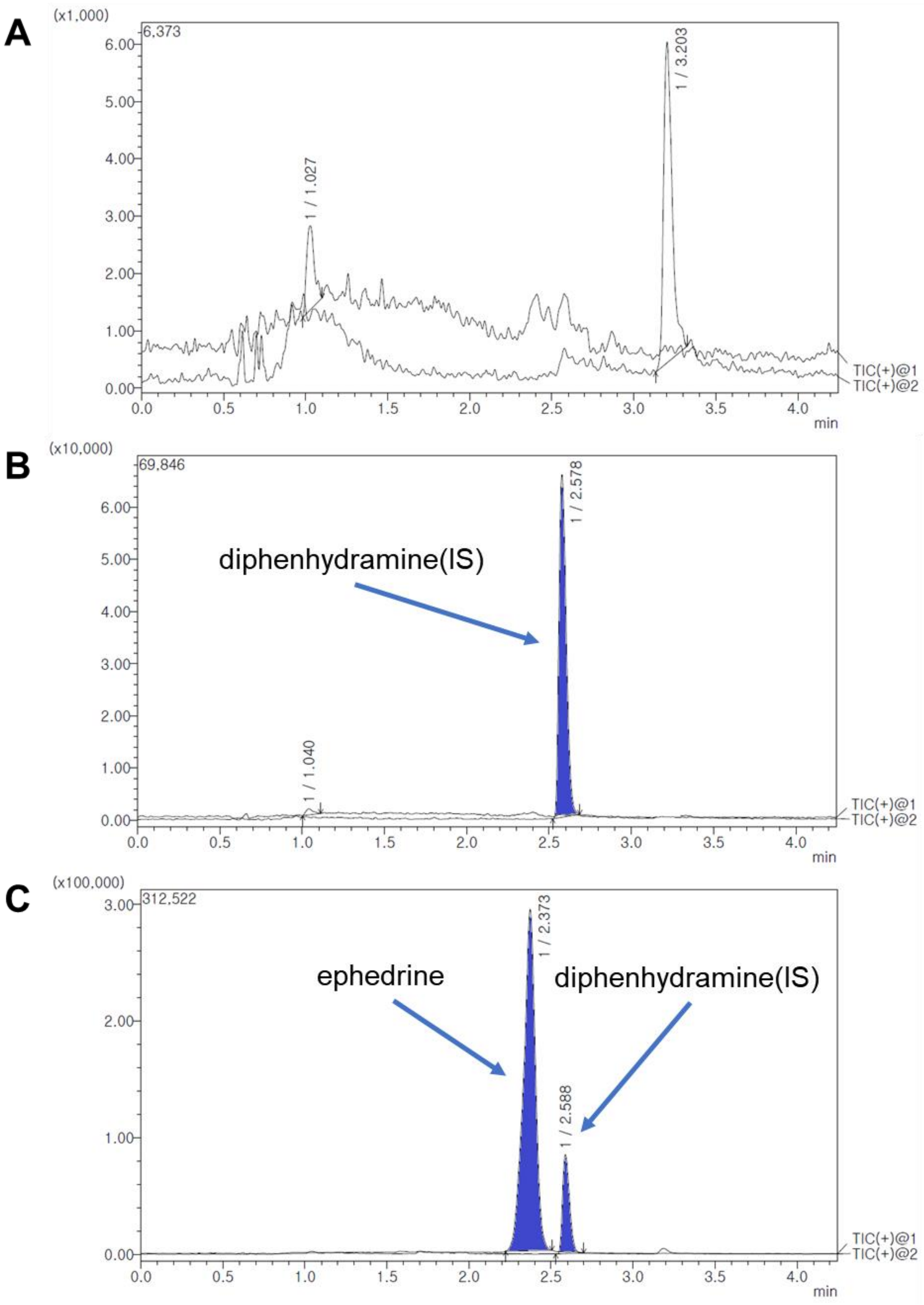

Figure S4. MRM chromatograms of ephedrine in blank plasma (**A**), zero plasma containing the IS (**B**), and a plasma sample at 0.75 h after the oral administration of a SCRT tablet (**C**).

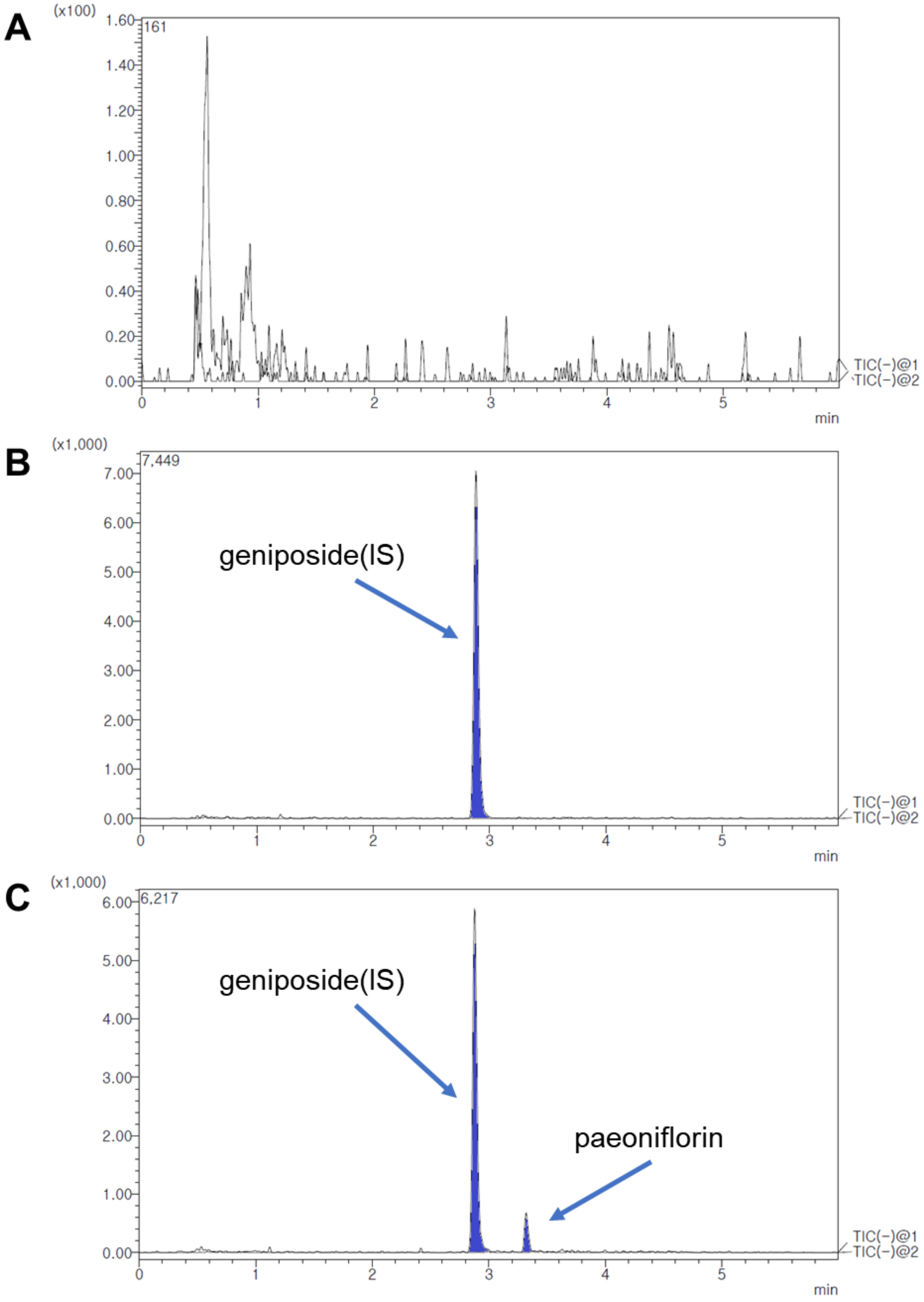

Figure S5. MRM chromatograms of paeoniflorin in blank plasma (**A**), zero plasma containing the IS (**B**), and a plasma sample at 0.75 h after the oral administration of a SCRT tablet (**C**).

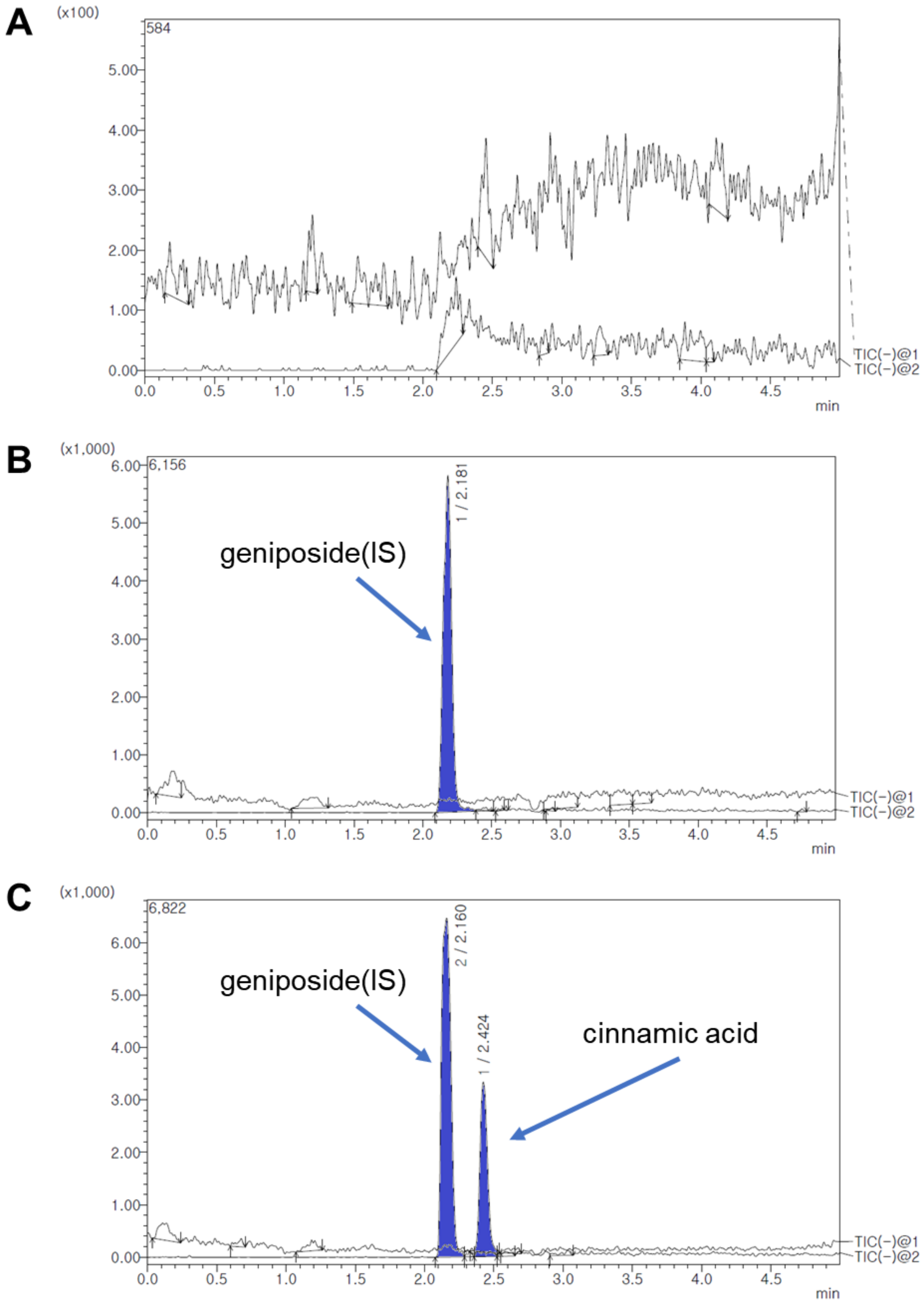

Figure S6. MRM chromatograms of cinnamic acid in blank plasma (**A**), zero plasma containing the IS (**B**), and a plasma sample at 0.75 h after the oral administration of a SCRT tablet (**C**).

References

1. Wan, H.; Pan, L.; Wang, Y.; Li, C.; Yu, L.; Zhou, H.; Wan, H.; He, Y. Pharmacokinetics of seven major active components of Mahuang decoction in rat blood and brain by LC–MS/MS coupled to microdialysis sampling. *Naunyn-Schmiedeberg's Arch. Pharmacol.* **2020**, *393*, 1559–1571.

2. Tang, Y.; Zheng, M.; Chen, Y.-L.; Chen, J.; He, Y. Pharmacokinetic effects of cinnamic acid, amygdalin, glycyrrhizic acid and liquiritin on ephedra alkaloids in rats. *Eur. J. Drug Metab. Pharmacokinet.* **2017**, *42*, 527–535.
3. Wang, J.-W.; Chiang, M.-H.; Lu, C.-M.; Tsai, T.-H. Determination the active compounds of herbal preparation by UHPLC–MS/MS and its application on the preclinical pharmacokinetics of pure ephedrine, single herbal extract of Ephedra, and a multiple herbal preparation in rats. *J. Chromatogr. B* **2016**, *1026*, 152–161.
4. Song, Y.; Su, D.; Lu, T.; Mao, C.; Ji, D.; Liu, Y.; Wei, B.; Fan, R. Differential pharmacokinetics and the brain distribution of morphine and ephedrine constitutional isomers in rats after oral administration with Keke capsule using rapid-resolution LC–MS/MS. *J. Sep. Sci.* **2014**, *37*, 352–359.
5. Wei, P.; Huo, H.-l.; Ma, Q.; Li, H.; Xing, X.; Tan, X.; Luo, J. Pharmacokinetic comparisons of five ephedrine alkaloids following oral administration of four different Mahuang–Guizhi herb-pair aqueous extracts ratios in rats. *J. Ethnopharmacol.* **2014**, *155*, 642–648.
6. Wang, C.; Yuan, J.; Zhang, L.L.; Wei, W. Pharmacokinetic comparisons of Paeoniflorin and Paeoniflorin-6′O-benzene sulfonate in rats via different routes of administration. *Xenobiotica* **2016**, *46*, 1142–1150.
7. Wang, X.; Ma, X.; Li, W.; Chu, Y.; Guo, J.; Li, S.; Wang, J.; Zhang, H.; Zhou, S.; Zhu, Y. Simultaneous determination of five phenolic components and paeoniflorin in rat plasma by liquid chromatography–tandem mass spectrometry and pharmacokinetic study after oral administration of Cerebralcare granule®. *J. Pharm. Biomed. Anal.* **2013**, *86*, 82–91.
8. Xu, C.-H.; Wang, P.; Wang, Y.; Yang, Y.; Li, D.-H.; Li, H.-F.; Sun, S.-Q.; Wu, X.-Z. Pharmacokinetic comparisons of two different combinations of Shaoyao-Gancao Decoction in rats: Competing mechanisms between paeoniflorin and glycyrrhetinic acid. *J. Ethnopharmacol.* **2013**, *149*, 443–452.
9. Jiang, F.; Zhao, Y.; Wang, J.; Wei, S.; Wei, Z.; Li, R.; Zhu, Y.; Sun, Z.; Xiao, X. Comparative pharmacokinetic study of paeoniflorin and albiflorin after oral administration of Radix Paeoniae Rubra in normal rats and the acute cholestasis hepatitis rats. *Fitoterapia* **2012**, *83*, 415–421.
10. Hwang, Y.-H.; Kim, T.; Cho, W.-K.; Jang, D.; Ha, J.-H.; Ma, J.Y. Food-and gender-dependent pharmacokinetics of paeoniflorin after oral administration with Samul-tang in rats. *J. Ethnopharmacol.* **2012**, *142*, 161–167.
11. Gan, P.; Zhong, M.; Huang, X.; Sun, M.; Wang, Y.; Xiao, Y.; Zeng, C.; Yuan, Q.; Liu, Z.; Zhou, H. Pharmacokinetic comparisons of albiflorin and paeoniflorin after oral administration of Shaoyao-Gancao-Tang and single herb Paeony decoction to rats. *Planta. Med.* **2012**, *78*, 237–243.
12. Liu, J.; Wang, J.-S.; Kong, L.-Y. Comparative pharmacokinetics of paeoniflorin in plasma of vascular dementia and normal rats orally administrated with Danggui-Shaoyao-San or pure paeoniflorin. *Fitoterapia* **2011**, *82*, 466–473.
13. Feng, C.; Liu, M.; Shi, X.; Yang, W.; Kong, D.; Duan, K.; Wang, Q. Pharmacokinetic properties of paeoniflorin, albiflorin and oxypaeoniflorin after oral gavage of extracts of Radix Paeoniae Rubra and Radix Paeoniae Alba in rats. *J. Ethnopharmacol.* **2010**, *130*, 407–413.
14. Wu, H.; Zhu, Z.; Zhang, G.; Zhao, L.; Zhang, H.; Zhu, D.; Chai, Y. Comparative pharmacokinetic study of paeoniflorin after oral administration of pure paeoniflorin, extract of Cortex Moutan and Shuang-Dan prescription to rats. *J. Ethnopharmacol.* **2009**, *125*, 444–449.
15. Wang, C.; Wang, R.; Cheng, X.; He, Y.; Wang, Z.; Wu, C.; Cao, J. Comparative pharmacokinetic study of paeoniflorin after oral administration of decoction of Radix Paeoniae Rubra and Radix Paeoniae Alba in rats. *J. Ethnopharmacol.* **2008**, *117*, 467–472.
16. Liu, Z.Q.; Zhou, H.; Liu, L.; Jiang, Z.H.; Wong, Y.F.; Xie, Y.; Cai, X.; Xu, H.X.; Chan, K. Influence of co-administrated sinomenine on pharmacokinetic fate of paeoniflorin in unrestrained conscious rats. *J. Ethnopharmacol.* **2005**, *99*, 61–67.
17. Takeda, S.; Isono, T.; Wakui, Y.; Matsuzaki, Y.; Sasaki, H.; Amagaya, S.; Maruno, M. Absorption and excretion of paeoniflorin in rats. *J. Pharm. Pharmacol.* **1995**, *47*, 1036–1040.
18. Guan, J.; Wang, L.; Jin, J.; Chang, S.; Xiao, X.; Feng, B.; Zhu, H. Simultaneous determination of calycosin-7-O-β-D-glucoside, cinnamic acid, paeoniflorin and albiflorin in rat plasma by UHPLC-MS/MS and its application to a pharmacokinetic study of Huangqi Guizhi Wuwu Decoction. *J. Pharm. Biomed. Anal.* **2019**, *170*, 1–7.
19. Ji, B.; Zhao, Y.; Yu, P.; Yang, B.; Zhou, C.; Yu, Z. LC-ESI-MS/MS method for simultaneous determination of eleven bioactive compounds in rat plasma after oral administration of Ling-Gui-Zhu-Gan Decoction and its application to a pharmacokinetics study. *Talanta* **2018**, *190*, 450–459.
20. Ji, B.; Zhao, Y.; Zhang, Q.; Wang, P.; Guan, J.; Rong, R.; Yu, Z. Simultaneous determination of cinnamaldehyde, cinnamic acid, and 2-methoxy cinnamic acid in rat whole blood after oral administration of volatile oil of Cinnamoni Ramulus by UHPLC-MS/MS: An application for a pharmacokinetic study. *J. Chromatogr. B* **2015**, *1001*, 107–113.
21. Zhao, L.; Xiong, Z.; Sui, Y.; Zhu, H.; Zhou, Z.; Wang, Z.; Zhao, Y.; Xiao, W.; Lin, J.; Bi, K. Simultaneous determination of six bioactive constituents of Guizhi Fuling Capsule in rat plasma by UHPLC–MS/MS: Application to a pharmacokinetic study. *J. Chromatogr. B* **2015**, *1001*, 49–57.
22. Basu, S.; Patel, V.B.; Jana, S.; Patel, H. Liquid chromatography tandem mass spectrometry method (LC–MS/MS) for simultaneous determination of piperine, cinnamic acid and gallic acid in rat plasma using a polarity switch technique. *Anal. Methods* **2013**, *5*, 967–976.
23. Li, P.; Zhang, Y.; Xiao, L.; Jin, X.; Yang, K. Simultaneous determination of harpagoside and cinnamic acid in rat plasma by high-performance liquid chromatography: Application to a pharmacokinetic study. *Anal. Bioanal. Chem.* **2007**, *389*, 2259–2264.
24. Chen, Y.; Ma, Y.; Ma, W. Pharmacokinetics and bioavailability of cinnamic acid after oral administration of Ramulus Cinnamomi in rats. *Eur. J. Drug Metab. Pharmacokinet.* **2009**, *34*, 51–56.
